# Machine learning assisted metamaterial-based reconfigurable antenna for low-cost portable electronic devices

**DOI:** 10.1038/s41598-022-16678-2

**Published:** 2022-07-19

**Authors:** Shobhit K. Patel, Jaymit Surve, Vijay Katkar, Juveriya Parmar

**Affiliations:** 1 Department of Computer Engineering, Marwadi University, Rajkot, Gujarat India; 2 Department of Electrical Engineering, Marwadi University, Rajkot, Gujarat India; 3 Department of Electronics and Communication Engineering, Marwadi University, Rajkot, Gujarat India; 4 Department of Mechanical and Materials Engineering, University of Nebraska-Lincoln, 1400 R St., Nebraska, 68588 USA

**Keywords:** Electrical and electronic engineering, Electronic properties and materials, Electronic devices, Metamaterials

## Abstract

Antenna design has evolved from bulkier to small portable designs but there is a need for smarter antenna design using machine learning algorithms that can meet today’s high growing demand for smart and fast devices. Here in this research, main focus is on developing smart antenna design using machine learning applicable in 5G mobile applications and portable Wi-Fi, Wi-MAX, and WLAN applications. Our design is based on the metamaterial concept where the patch is truncated and etched with a split ring resonator (SRR). The high gain requirement is met by adding metamaterial superstrates having thin wires (TW) and SRRs. The reconfigurability is achieved by adding three PIN diode switches. Multiple designs have been observed by adding superstrate layers ranging from one layer to four layers with interchanging TWs and SRRs. The TW metamaterial superstrate design with two layers is giving the best performance in gain, bandwidth, and the number of bands. The design is optimized by changing the path’s physical parameters. To shrink simulation time, Extra Tree Regression based machine learning model is used to learn the behavior of the antenna and predict the reflectance value for a wide range of frequencies. Experimental results prove that the use of the Extra Tree Regression based model for simulation of antenna design can cut the simulation time, resource requirements by 80%.

## Introduction

Antenna development has evolved from bulkier designs to low weight portable designs. The miniaturized design having low weight and size is required to be used in portable devices. The miniaturized antenna designs have a drawback of a lower gain which needs to be investigated. This drawback can be overcome by incorporating metamaterials in the antenna design^1^. There are several attempts to improve the gain by incorporating metamaterials, applying meandering, etc. but there is still scope for improving this further using similar techniques. Furthermore, the reconfiguration is required to be applicable in multiple applications like WiMAX, WLAN, 5G, etc.^2,3^. This reconfiguration can be realized by applying switching with RF MEMS switches, PIN diodes, etc.^4,5^.

Metamaterials are artificial materials that give properties like negative permittivity and permeability that improve several parameters of the antenna^6^. SRR and TWs are the two effective structures used widely for incorporating metamaterials in antenna design^7^. A complementary SRR is also used to etch the ground plane which improves different parameters of the patch antenna^8^. Yuan et al. presented a chirality-assisted phase with promising advances in reconfigurable beam antennas^9^. Zhang and co-authors developed two vortex beam generators with potential application in orbital angular momentum communication systems^10^. Metamaterial antennas are applicable in several applications like wi-fi, WLAN, Wi-MAX, wearable devices, etc. Metamaterials are also useful in achieving beam scanning, improving gain, reducing the size, multifrequency operation, etc. Metamaterial superstrate is added to a simple microstrip patch antenna to improve the gain of the antenna^11^. Metamaterial loaded Vivaldi antenna with its high gain capability can be employed for imaging applications^12^. Metamaterial loaded antennas are used in designing wearable devices^13,14^. Radiation beam scanning is very important in designing antennas and this scanning can be achieved by metamaterial antenna^15^. Antenna size can also be reduced by loading metamaterials in the antenna^16^.

Superstrate metamaterial can be used to improve the gain of the antenna. Superstrates are stacked above the microstrip patch one after another to improve the gain and enhanced the radiation behavior of the antenna. Saravanan et al. presented a metamaterial assisted superstrate antenna for modern wireless applications that achieves the highest gain of 7.94 dB with a reflection coefficient of − 28.64 dB at 2.4 GHz^17^. Patel and co-authors fabricated a microstrip based structure with enhanced gain that can be utilized as a unit block of a radar system for surveillance applications^18^. Ojo et al. reported a MIMO array antenna for enhancing gain and bandwidth and the bandwidth improved by 12.45%^19^. Sumathi and co-authors designed a metamaterial superstrate based microstrip patch antenna with pin diodes as a switching mechanism for applications in wireless network devices for C/X/Ku-band^20^.

Switching can be applied to antenna structures by incorporating PIN diodes. These PIN diodes can be ‘on’ or ‘off’ to achieve the reconfiguration in frequency and radiation pattern. Because of their low insertion loss, cheap cost, strong switching characteristics, and high isolation, PIN diodes are the best choice for reconfigurable antennas^21^. To provide reconfigurability, a PIN diode may be used to link different portions of the patch to modify the slot’s length. To obtain a better gain response, a different shaped patch is also employed^22,23^. Because of its smaller size, better gain, and similar sort of radiation pattern, the reconfigurable antenna is more commonly utilized than the multiband antenna^24^. Two PIN diodes are utilized to create a reconfigurable antenna that provides high gain, low return losses, and an excellent radiation pattern for WiMax/WiFi applications^25^. The results of pixel antennas are compared and studied, resulting in a satisfactory frequency response^26,27^. To generate a suitable pattern, the single PIN diode is intended to act in two distinct modes at different frequencies. They're employed in elements like cognitive radio systems, satellite applications, biomedical applications, and filters, among other applications^28,29^. By turning ON/OFF slots, the radiation pattern may be changed. Reconfigurable antennas are small and simple to manufacture^30^.

Machine learning (ML) can be applied to predict or forecast the behavior of different parameters of electronic and photonic systems. ML can also be used to observe, predict, and forecast the reflectance response of the photonic devices^31–34^. Misilmani reviewed ML applications for antenna designs utilizing regression models^35^. Bessa and co-authors also applied ML for metamaterials design for adaptation of target characteristics, base material selection, and manufacturing process^36^. Deringer reports how ML enables access to essential simulations that achieve equal levels of precision but are hundreds of times faster^37^.

In this paper, we developed a new ML based antenna design that not only gives reconfiguration, high gain, and multiband response but also predicts the behavior of the antenna using ML algorithms. We have presented the design and its modeling in Sect. 2. The measured and simulation results are presented and compared with fabrication results in Sect. 3. The ML behavior prediction to shrink the simulation time necessity is available in Sect. 4 following the concluding remarks in Sect. 5.

## Design and modelling

**Figure 1:**
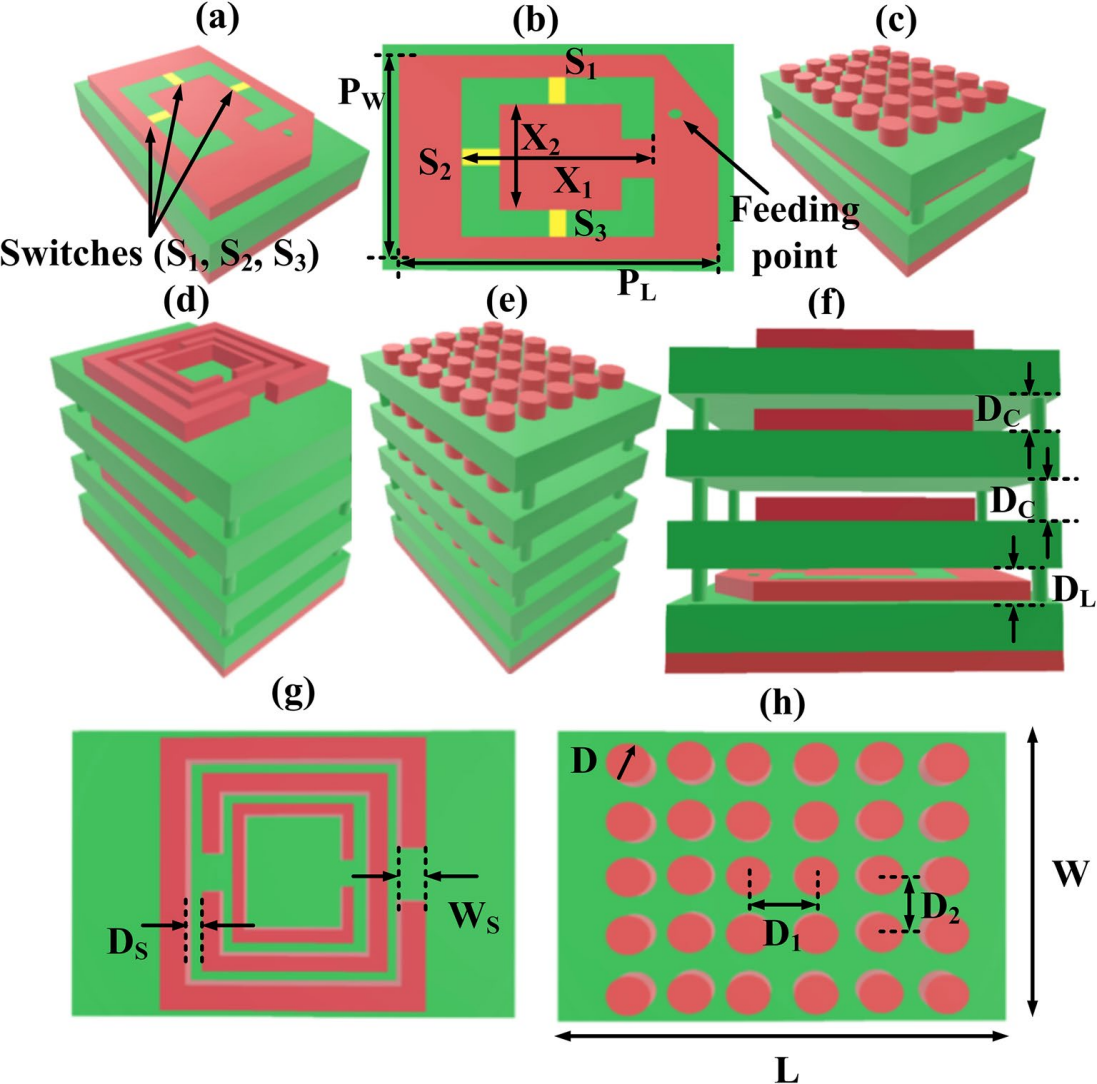
Various structures of proposed antenna designs **(a)** patch structure with switches formulated with the help of pin diodes, **(b)** top view of patch structure **(c)** 3D view of 6 × 5 TW double layer structure followed by a patch layer, **(d)** 3D view of three layer SRR followed by a patch layer, **(e)** 3D view of 6 × 5 TW four layer structure followed by a patch layer, **(f)** front view of three layer SRR followed by a patch layer, **(g)** top view of SRR, **(h)** top view of 6 × 5 TW layer.

We have proposed various superstrate antenna structures and their representation is reported in Fig. 1. Multiple structures including a 6 × 5 TW single layer structure, 6 × 5 TW double layer structure, 6 × 5 TW triple layer structure, 6 × 5 TW four layer structure have been designed. The base layer in all these designs is common which is a split ring based patch structure. The 3D view and overhead view of the split ring based patch structure with switches S_1_, S_2_, and S_3_ are available in Fig. 1a and Fig. 1b. The PIN diode model is presented in supplementary material as Fig. S17 and their properties are taken from^20^. The feeding point is also demonstrated in Fig. 1b. As shown in Fig. 1a, the split ring based patch structure and ground layer of the patch antenna are fabricated using copper material and the substrate is of Fr_4_ material. The thickness of the ground layer and SRR structure is 0.35 mm. The thickness of the Fr_4_ substrate layer is 1.5 mm. The overall length and the width of the structure, L and W are 84 mm and 64 mm, respectively. The length and width of a patch antenna, P_L,_ and P_W_ are 74 mm and 54 mm, respectively. The inner and outer length of the split ring patch structure, X_1_ and X_2_ is kept at 25 mm and 15 mm, respectively. The split ring gap of the SRR is kept at 3 mm. The distance between the patch layer and the first superstrate layer, D_L_ is 10 mm and then the distance between two consecutive superstrate layers, D_C_ is 6 mm as presented in Fig. 1f. The 6 × 5 TW double layer and 6 × 5 TW four layer structure are represented in Fig. 1c,e, respectively. The diameter, D of the circle TW is 2.5 mm as illustrated in Fig. 1h. Alongside this, we have also designed the three layer SRR structure followed by the split ring based patch antenna as reported in Fig. 1d. The dimension of substrate thickness and patch structure is kept constant. The width of SRR, W_S_ is 3 mm, and the distance between two consecutive SRR, D_S_ is 3 mm as shown in Fig. 1g. The split ring gap and thickness of the SRR are kept at 3 mm and 0.35 mm, respectively. The distance between two consecutive horizontal and vertical TW is 12 nm and 10 nm, respectively. To support the multilayer 3D printed hollow boxes of corresponding thickness are fabricated and utilized for testing purposes. For these support boxes, plastic-based material is selected to avoid meddling in the radiating field of the antenna. To accomplish frequency reconfigurability, we employed a PIN diode as a switch to modify the charge distribution in the patch. For the excitation of the proposed design, a coaxial feed is employed. The three PIN diode joins two pieces of the patch area and is positioned in the middle of the c shape lines.

The reflectance coefficient (S_11_) and transmittance coefficient (S_21_) play a major role to determine the wave impedance (z), and refractive index (n) as described in Eqs. (1–2). Metamaterial permittivity ($\varepsilon$) and permeability ($\mu$) equations are provided in Eqs. (3–4) derived from the impedance and refractive index.

1 $$z= \pm \sqrt{\frac{{(1+{S}_{11})}^{2}-{{S}_{21}}^{2}}{{(1-{S}_{11})}^{2}-{{S}_{21}}^{2}}}$$

2 $$n=\frac{1}{kd}{\mathrm{cos}}^{-1}\left[\frac{1}{2{S}_{21}}\left(1-{{S}_{11}}^{2}+{{S}_{21}}^{2}\right)\right]$$

3 $$\varepsilon =\frac{n}{z}$$

4 $$\mu =nz$$

where wave number is represented by k, the thickness of the layer is denoted by d.

## Results and discussion

### Simulation result analysis

**Figure 2:**
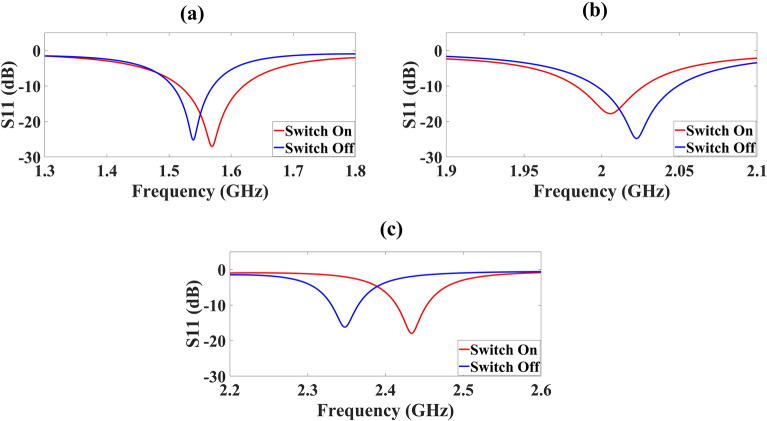
Simulated results of reflectance response (S_11_) for switching scenarios of three pin diodes for 1.9 GHz to 5.4 GHz **(a)** for 1.3 GHz to 1.8 GHz, the frequency reconfigures from 1.539 GHz to 1.569 GHz **(b)** for 1.9 GHz to 2.1 GHz, the frequency reconfigures from 2.006 GHz to 2.022 GHz **(c)** for 2.2 GHz to 2.6 GHz, the frequency reconfigures from 2.348 GHz to 2.434 GHz.

Our design is based on the metamaterial concept where the patch is truncated and etched with an SRR that is the common base layer for all six antenna superstrate structures. The high gain requirement is met by adding metamaterial superstrates having TWs and SRRs. The metamaterial properties of the single layer 6 × 5 TW pattern are presented in supplementary material as Fig. S18 and it is visible that the refractive index of this layer achieves the negative refractive index validating metamaterial characteristics. The reconfigurability is achieved by adding three PIN diode switches S_1_, S_2_, and S_3_ with their plots in Fig. 2 for two modes (ON and OFF). The developed antenna is observed for 1–7 GHz and for simplicity and clear reconfiguration analysis, we have divided the results into three frequency bands as we can observe in Fig. 2a–c. From 1.3 to 1.8 GHz for switch off mode the highest reflectance response of − 25.27 dB at 1.539 GHz is achieevd, and for switch on mode the frequency reconfigures to 1.569 GHz, and the reflectance response is also enhanced to − 27.11 dB as provided in Fig. 2a. From 1.9 to 2.1 GHz, we achieve the reflectance of − 17.79 dB at 2.006 GHz for switch on mode, and the enhanced reflectance of − 24.82 dB at reconfigured frequency of 2.022 GHz is achieved for switch off mode as reported in Fig. 2b. For 2.2–2.6 GHz for switch off mode, we achieved the highest reflectance response of − 16.23 dB at 2.348 GHz, and for switch on the mode the frequency reconfigures to 2.434 GHz, and the reflectance response is also enhanced to − 17.99 dB as shown in Fig. 2c.

**Figure 3:**
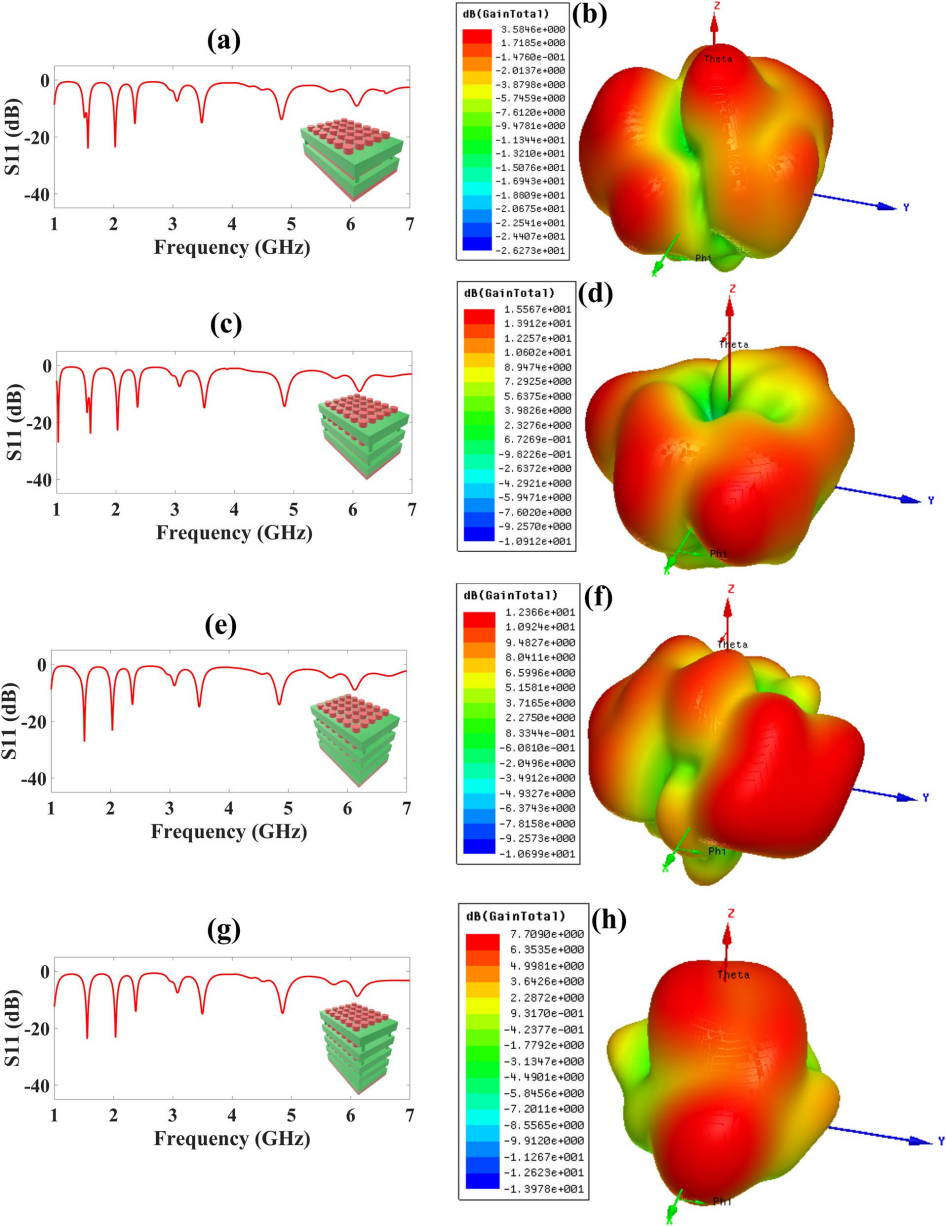
Simulated results of reflectance response (S_11_) and 3D Gain polar plot for various superstrate structures **(a)** for 6 × 5 TW single layer superstrate structure, five frequency bands are attained, **(b)** 6 × 5 TW single layer superstrate structure with gain 3.58 dB, **(c)** for 6 × 5 TW double layer superstrate structure, six frequency bands are attained, **(d)** 6 × 5 TW double layer superstrate structure with gain 15.57 dB, **(e)** for 6 × 5 TW triple layer superstrate structure, five frequency bands are attained, **(f)** 6 × 5 TW triple layer superstrate structure with gain 12.37 dB, **(g)** for 6 × 5 TW four layer superstrate structure, 5 frequency bands are attained. **(h)** 6 × 5 TW four layer superstrate structure with gain 7.71 dB.

For this research work, we have carried out the simulation of six structures of antenna. Multiple designs have been observed by adding superstrate layers ranging from one layer to four layers with interchanging TWs and SRRs. The first structure is a 6 × 5 TW single layer placed over a base layer of SRR based patch structure and the other three designs are designed by consecutively increasing the layer of 6 × 5 TW pattern up to four layers one by one keeping the base layer same. The reflectance response of 6 × 5 TW with one layer to four layer superstrate structure is presented in Fig. 3a,c,e,g.

The 6 × 5 TW single layer superstrate structure’s reflectance response is reported in Fig. 3a and as we can observe we obtain five bands and the highest reflectance response attained is the − 24.05 dB at 1.568 GHz with the highest bandwidth of 100 MHz. For the 6 × 5 TW double layer superstrate structure the reflectance of − 27.07 dB at 1.07 GHz with the six frequency bands and highest bandwidth of 108 MHz as reported in Fig. 3c. The 6 × 5 TW triple layer superstrate structure’s reflectance is reported in Fig. 3e and as we can observe we attained five bands and the highest reflectance response attained is the − 27.18 dB at 1.561 GHz with the highest bandwidth of 105 MHz. For the 6 × 5 TW four layer superstrate structure the reflectance of − 23.74 dB at 1.555 GHz with the five frequency bands and the highest bandwidth of 105 MHz as provided in Fig. 3f.

We have also carried out the gain polar plot for these four designs reported in Fig. 3b,d,f,h. The highest gain of 3.58 dB, 15.57 dB, 12.37 dB, and 7.71 dB for 6 × 5 TW single layer superstrate, 6 × 5 TW double layer superstrate, 6 × 5 TW triple layer superstrate, and 6 × 5 TW four layer superstrate structures, respectively and corresponding 3D gain polar plot are presented in Fig. 3b,d,f,h. So, we can conclude from reflectance response, no. of bands attained, and 3D gain polar plot results, that the 6 × 5 TW double layer superstrate structure performs comparatively better than other structures.

**Figure 4:**
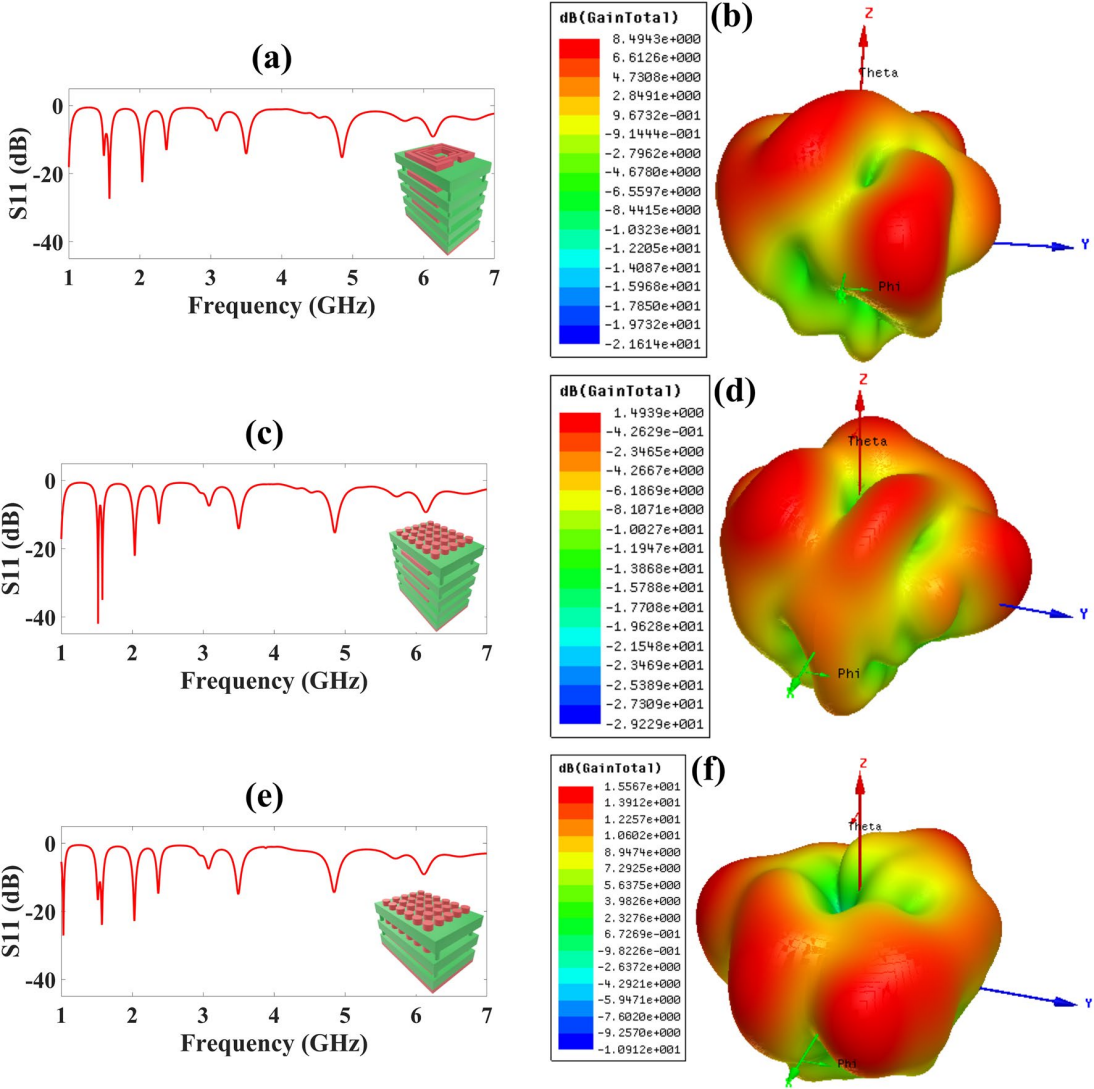
Simulated results of reflectance response (S_11_) and 3D Gain polar plot for various superstrate structures **(a)** SRR four layer superstrate structure, five frequency bands are attained, **(b)** for SRR four layer superstrate structure highest gain of 8.49 dB is attained, **(c)** for SRR three layer combined with 6 × 5 TW single layer as a top layer, we attain five frequency bands, **(d)** for SRR three layer combined with 6 × 5 TW single layer as a top layer, highest gain of 1.49 dB is attained, **(e)** for 6 × 5 TW double layer superstrate structure, six frequency bands are attained. **(f)** for 6 × 5 TW double layer superstrate structure, highest gain of 15.57 dB is attained.

Furthermore, to obtain a more optimized structure, we simulated several more designs and compared the results with the 6 × 5 TW double layer superstrate structure. We have simulated the four layer SRR followed by an SRR based patch layer and a 6 × 5 TW single layer structure placed upon three layer SRR followed by an SRR based patch layer and the corresponding reflectance response is represented in Fig. 4a,c,e. For four layer SRR followed by an SRR based patch layer, we attained five frequency bands with the highest reflectance of − 27.40 dB at 1.573 GHz and the highest bandwidth of 105 MHz as reported in Fig. 4a. For 6 × 5 TW single layer structure placed upon three layer SRR followed by an SRR based patch layer, we attained five frequency bands with the highest reflectance of − 41.97 dB at 1.518 and with the highest bandwidth of 110 MHz GHz as reported in Fig. 4c. For the 6 × 5 TW double layer superstrate structure the reflectance of − 27.07 dB at 1.07 GHz with the six frequency bands and highest bandwidth of 108 MHz as reported in Fig. 4e.

We have also carried out the gain polar plot for these three designs and the results are reported in Fig. 4b,d,f. The highest gain of 8.49 dB, 1.49 dB, and 15.57 dB for four layers SRR followed by an SRR based patch layer, 6 × 5 TW single layer structure placed upon three layer SRR followed by an SRR based patch layer, and 6 × 5 TW double layer superstrate, respectively and corresponding 3D gain polar plot are presented in Fig. 4b,d,f. So, we can conclude from reflectance response, no. of bands attained, and 3D gain polar plot results, that the 6 × 5 TW double layer superstrate structure performs comparatively better than SRR structures, too. Furthermore, we have carried out the variation of structural parameters of SRR based patch structure and corresponding results are presented in Figs. S1 and S2.

### Fabrication procedure and analysis

**Figure 5:**
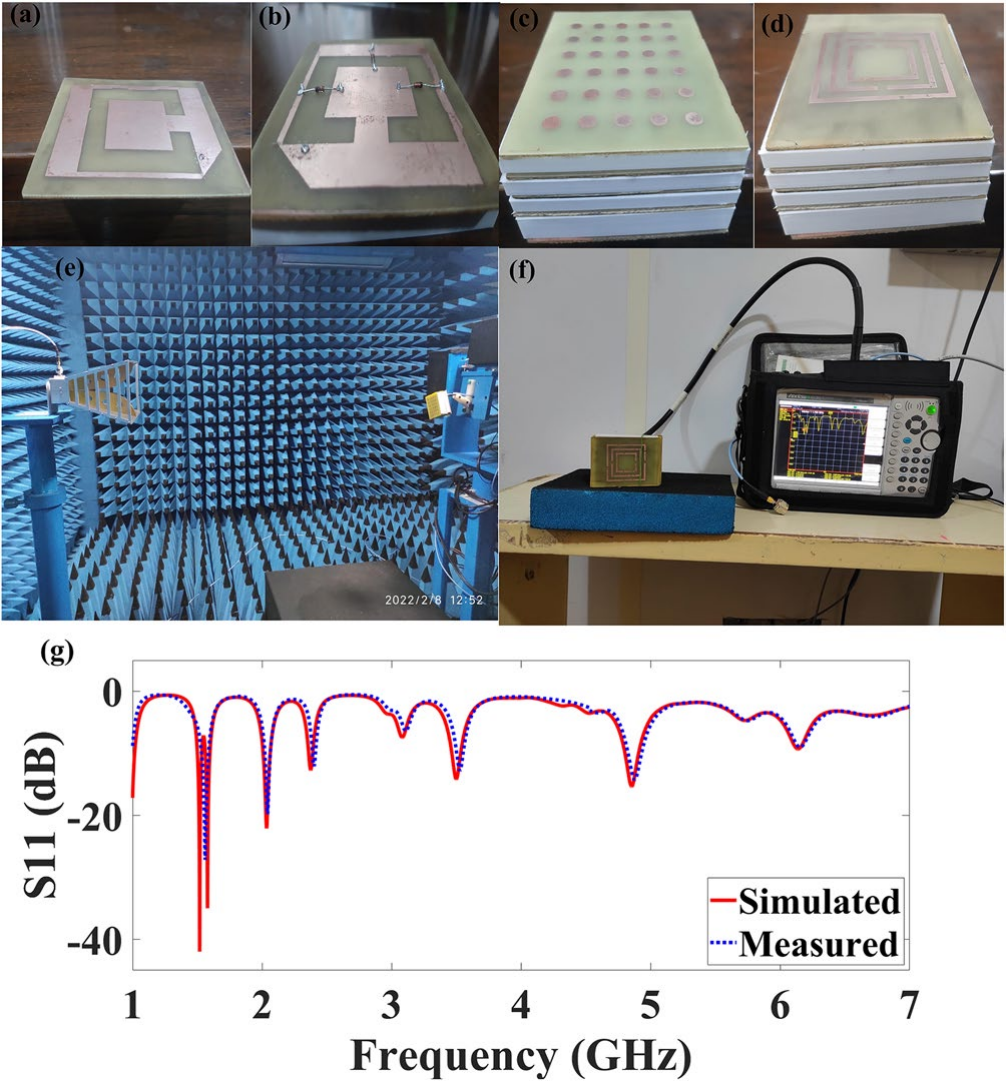
Fabricated structure and testing through the anechoic chamber **(a)** fabricated SRR based patch structure without pin diodes, **(b)** fabricated SRR based patch structure with pin diodes for switching mechanism to attain reconfiguration, **(c)** fabricated structure of 6 × 5 TW double layer superstrate structure placed upon SRR based patch structure separated using the 3D printed white hollow box of various thickness**, (d)** fabricated structure of four layer SRR superstrate structure placed upon SRR based patch structure separated using 3D printed white hollow box of various thickness, **(e)** antenna testing facility of anechoic chamber, **(f)** Reflectance response observation of four layer SRR superstrate structure placed upon SRR based patch structure **(g)** Comparison of simulated and measured results for SRR three layer structure combined with 6 × 5 TW single layer as a top layer, we attain five frequency bands.

To validate the simulation results, we have fabricated all the antenna structures and corresponding antenna structures with testing facilities, and comparison results are reported in Fig. 5. To fabricate the split ring-based patch structure we have utilized the Fr_4_ copper-clad laminated double side PCB sheet and split ring structure and feed is etched by removing the excessed copper material from the top layer using the FeCl_3_ solution. The sheet was placed in FeCl_3_ solution for half an hour and the patch structure is ready in the same manner rest of the antenna structures are fabricated using the Fr_4_ copper-clad laminated single side PCB. To fabricate a superstrate structure we required several support boxes to place between two consecutive superstrate layers and these were printed using the 3D printer for two thickness values as the thickness between the patch layer and first superstrate layer and two consecutive superstrate structures is different. The fabricated structure of SRR based patch layer without pin diodes and with pin diodes as a switching mechanism to attain reconfiguration is presented in Fig. 5a,b, respectively. Figure 5c illustrates the fabricated structure of 6 × 5 TW double layer superstrate structure placed upon SRR based patch structure separated using a 3D printed white hollow box of various thicknesses while Fig. 5d represents the fabricated structure of four layer SRR superstrate structure placed upon an SRR based patch structure separated using the 3D printed white hollow box of various thicknesses. The antenna was tested by placing it in the anechoic chamber as illustrated in Fig. 5e. The reflectance response of four layer SRR superstrate structure placed upon the SRR based patch structure is observed with the help of a vector network analyzer and the corresponding results are presented in Fig. 5f.

The comparison of simulated results with measured results for SRR three layer structure combined with a 6 × 5 TW single layer as a top layer is presented in Fig. 5g. It is visible from Fig. 5g that the measured result is identical to simulated results and verifies the simulation results. Furthermore, a comparison study to compare the proposed antenna structures with previously published work is also carried out and presented in supplementary Table ST1. It is quite clear that the proposed antenna structure of 6 × 5 TW double layer structure performs remarkably in terms of reflectance response, no. of bands, bandwidth, and gain. We have developed a smart antenna design using machine learning that is applicable in 5G mobile applications and portable Wi-Fi, Wi-MAX, and WLAN applications. The narrow band antennas are employed for various applications that include L band applications for satellite communication, digital audio broadcasting, amateur satellite up-link communications, 5G communication.

## Reflectance prediction using extra tree regression

This section briefly describes the need of using regression models during the simulation process and explains how regression models can be used to cut the time and resource requirements by 80% while simulating the efficiency of antenna design.

### Need of regression methods

Researchers utilize regression analysis to find the value of dependent parameter(s) using the value(s) of independent parameter(s)^38–41^. While simulating antenna design, frequency is an independent parameter, while reflectance value is a dependent parameter. Simulating the experimental design requires a significant amount of time and resources. The increase in complexity of the experimental design necessitates more time and resources. While simulating the efficiency of an antenna, it must be evaluated for a wide variety of frequency values. As the testing range expands, the simulation resource demand expands as well. As a result, the cost of modeling and experimentation rises. ML based regression analysis methodologies can be used to solve this problem using the following three steps.

*Step 1:* Simulate the design of the antenna using a higher stepsize value for frequency.

*Step 2:* Train the machine learning based regression model using simulated data.

*Step 3:* Predict the reflectance values of intermediate frequencies using the trained regression model.

With an increase in the step size value of frequency, simulation time and resource necessities are reduced. R Square Score (R^2^S), Mean Absolute Percentage Error (MAPE), Mean Squared Error (MSE), and Adjusted R Square Score (AR^2^S) are commonly used criteria to quantity the prediction correctness of trained regression model. Formulas for computing these metrics are indicated in Eqs. (5–8).

5 $$MSE= \frac{1}{N}\sum_{i=1}^{N}{({Actual\,Target\,Value}_{i}-{Predicted\,Target\,Value}_{i})}^{2}$$

6 $$MAPE=\frac{1}{n}\sum_{i=1}^{n}\left\lfloor\frac{{Actual\,Target\,Value}_{i}-{Predicted\,Target\,Value}_{i})}{{Actual\,Target\,Value}_{i}}\right\rfloor *100$$

7 $${R}^{2}S= 1- \frac{\sum_{i=1}^{N}{({Predicted \,Target\,Value}_{i}-{Actual\,Target\,Value}_{i})}^{2}}{\sum_{i=1}^{N}{({Actual\,Target}_{i}- Average\, Target\,Value )}^{2}}$$

8 $$A{R}^{2}S= 1-\left[\frac{\left(1-{R}^{2}\right)*(N-1)}{N-K-1}\right]$$

Here ‘N’ is a number of data points used to test the regression model and ‘K’ is a number of independent parameters employed to predict the value of the target parameter.

### Regression analysis using extra tree regression model (ExTRM)

A binary recursive partitioning algorithm is used to build the Regression Tree. Each recursive step is used to find a data point in the independent parameter, where splitting the data set into two halves minimizes the Mean Square Error in regression analysis. To improve the accuracy of its predictions, the Regression Tree may need to be pruned or trimmed.

### Extremely randomized trees (extra tree) regression

This algorithm creates a collection of ‘M’ number of unpruned Regression Trees RT_1_, ... RT_M_. Unlike Regression Tree, this technique chooses the cutoff point at random and grows all regression trees using the entire training dataset. As indicated in Eq. (9), the output of all Regression Trees is blended using arithmetic averaging.

9 $$Predicted\,Value= \sum_{j=1}^{M}{RT}_{j}(x)$$

Here, x is the value of an independent parameter.

### Experiment design for reflectance value prediction using ExTRM

The experiments are carried out using data obtained by simulating the antenna design presented in Sect. 2. T.S.-60, T.S.-70, T.S.-80, and T.S.-90 are four Test Scenarios (T.S.) that are used to verify how much simulation time and resource necessities may be reduced using a regression analysis approach. In Test scenario T.S.-P, (100-P)% simulated data points are selected using a uniform random selection strategy to train the ExTRM, while the remaining P% simulated data points are used to quantify the trained ExTRM's prediction correctness. The number of data points utilized to train and quantify the ExTRM during various Test Scenarios is detailed in Supplementary Table ST2.

### Experimental results for prediction using ExTRM

**Figure 6:**
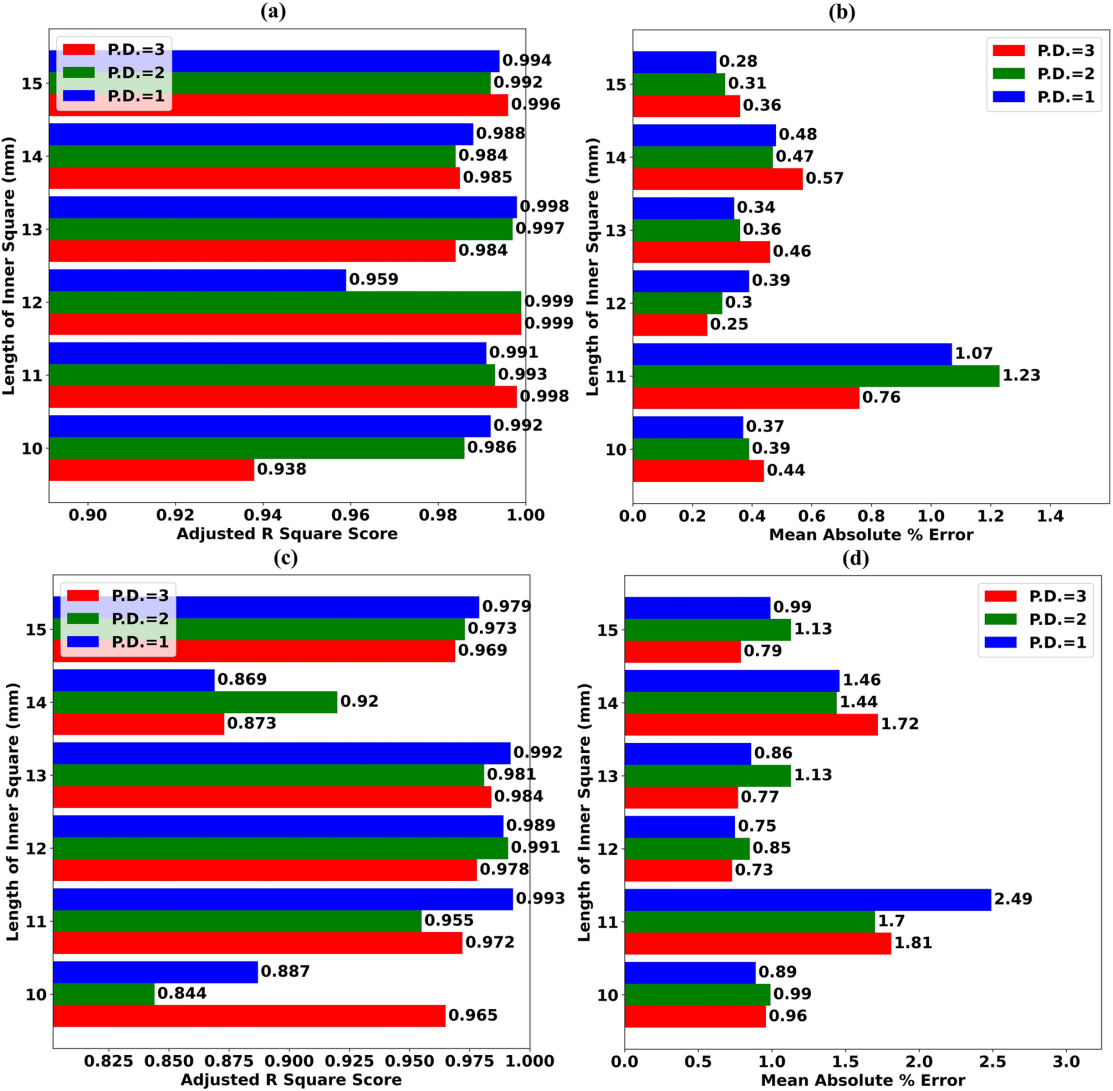
(**a**) AR^2^S of ExTRMs obtained for assorted values of Length of Inner Square (LIS) during Test Scenario (T.S.-80) (**b**) MAPE of ExTRMs for assorted values of LIS during Test Scenario (T.S.-80) (**c**) AR^2^S of ExTRMs obtained for assorted values of LIS during Test Scenario (T.S.-90) (**d**) MAPE of ExTRMs for assorted values of LIS during Test Scenario (T.S.-90).

100 Regression trees are used to create ExTRMs for experimentation. The AR^2^S of ExTRMs obtained for various values of Inner Square Length during Test Scenario T.S.-80 is shown in Fig. 6a.

The MAPE of ExTRMs for various values of Inner Square Length during Test Scenario T.S.-80 is depicted using a comparative bar chart in Fig. 6b. When ExTRMs are trained using first degree polynomial features (PF), an AR^2^S greater than 0.95 is obtained for all values of Inner Square Length, as shown in Fig. 6a. In addition, the MAPE of the ExTRMs is less than 0.5% for all values of Inner Square Length when the model is trained using first-degree PFs, except for the Inner Square length of 11 mm, as shown in Fig. 6b. It's roughly 1.0% in that situation.

**Figure 7:**
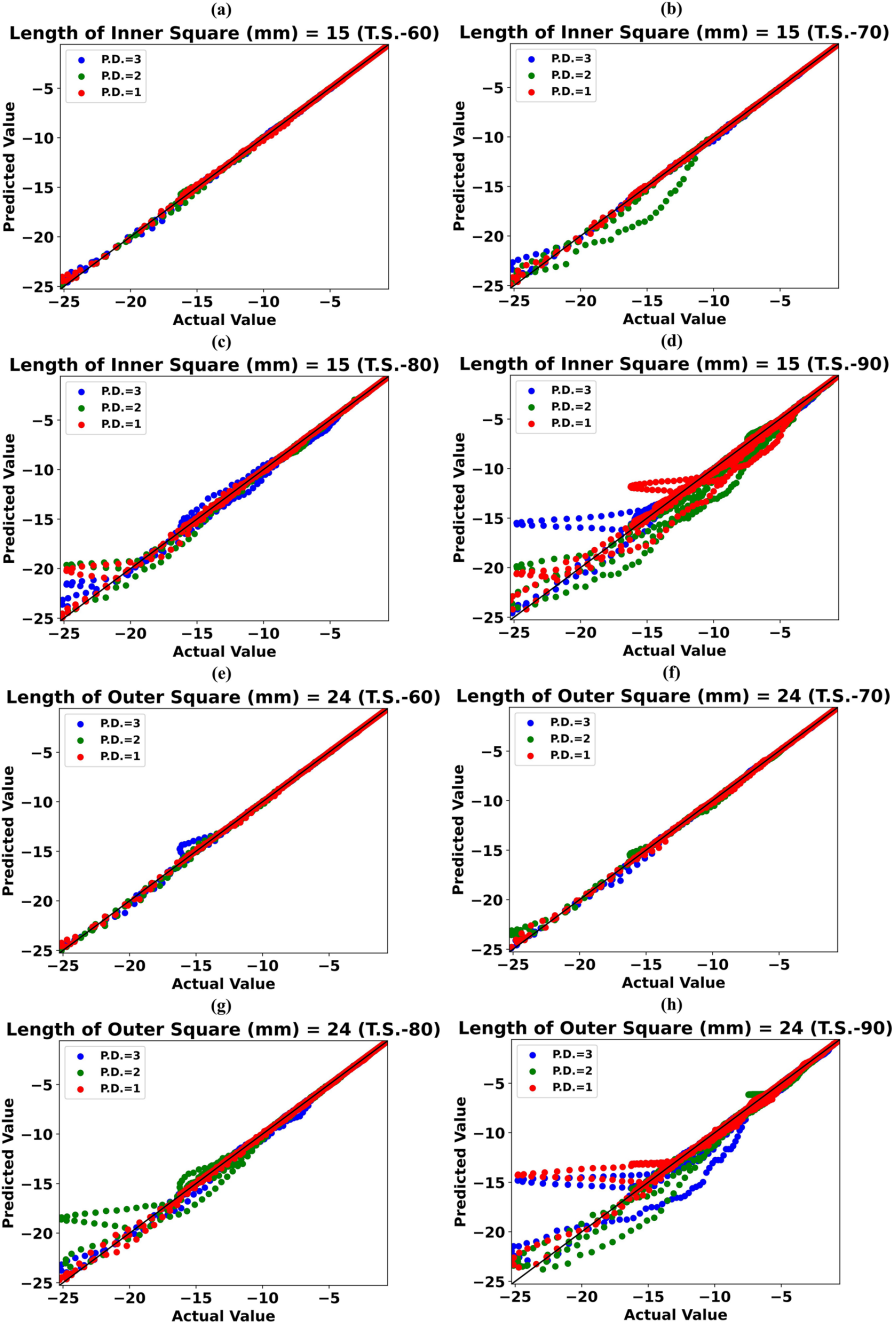
Scattergram of the Predicted value of reflectance vs Simulated value of reflectance for (**a**) Length of Inner Square (LIS) = 15 mm during T.S.-60 (**b**) LIS = 15 mm during T.S.-70 (**c**) LIS = 15 mm during T.S.-80 (**d**) LIS = 15 mm during T.S.-90 (**e**) Length of Outer Square (L0S) = 24 mm during T.S.-60 (**f**) L0S = 24 mm during T.S.-70 (**g**) L0S = 24 mm during T.S.-80 (**h**) L0S = 24 mm during T.S.-90.

Figure 7a–d shows scattergrams of predicted reflectance values vs. simulated reflectance values for Inner Square Length of 15 mm during Test Scenarios T.S.-60, T.S.-70, T.S.-80, and T.S.-90, respectively. Even though only 20% of the simulated data is utilized to forecast the reflectance value for the remaining 80% frequencies, the ExTRM can predict these values with high precision, as shown in Fig. 7c. The same cannot be said for Test Scenario T.S.-90. Supplementary Figs. (S3–S7) shows scattergrams for Inner Square lengths of 10–14 mm in 1 nm uniform increment, respectively. As a result, we can conclude that using the ExTRM during antenna design simulation for various values of Inner Square Length can cut simulation requirements by 80%.

The AR^2^S of ExTRMs obtained for various values of Inner Square Length during Test Scenario T.S.-90 is shown in Fig. 6c. The MAPE of ExTRMs for various values of Inner Square Length during Test Scenario T.S.-90 is depicted using a comparative bar chart in Fig. 6d. When ExTRMs are trained using first/second/third degree PFs, an AR^2^S of greater than 0.9 cannot be obtained for all values of Inner Square Length, as shown in Fig. 6c. In addition, the MAPE of the ExTRM is significantly more than 1.0% for some values of Inner Square Length, as shown in Fig. 6d. As a result, we can conclude that using the ExTRM during antenna design simulation for various values of Inner Square Length cannot cut simulation requirements by 90%.

**Figure 8:**
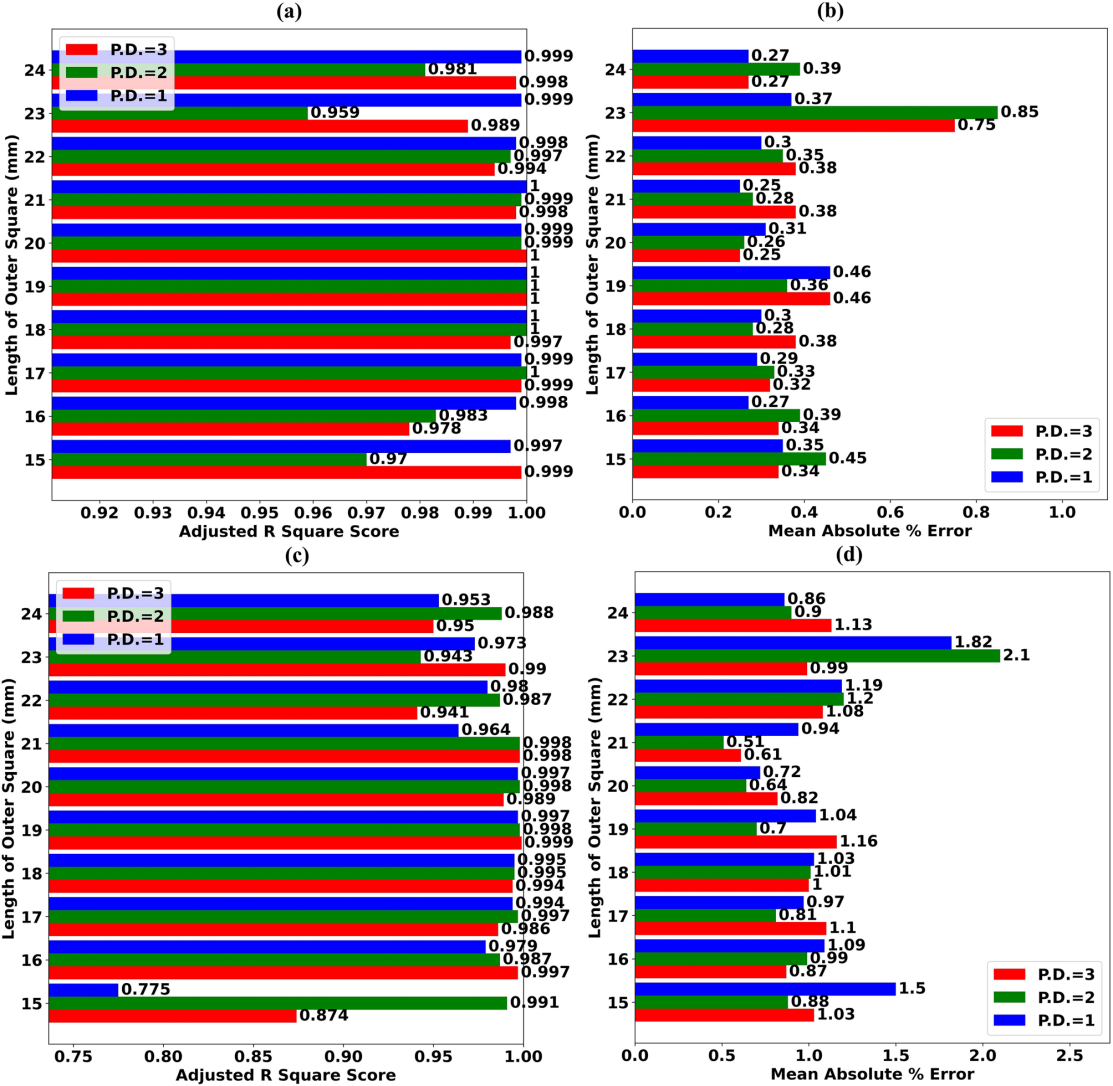
(**a**) AR^2^S of ExTRMs obtained for assorted values of Length of Outer Square (LOS) during Test Scenario (T.S.-80) (**b**) MAPE of ExTRMs for assorted values of LOS during Test Scenario (T.S.-80) (**c**) AR^2^S of ExTRMs obtained for assorted values of LOS during Test Scenario (T.S.-90) (**b**) MAPE of ExTRMs for assorted values of LOS during Test Scenario (T.S.-90).

The AR^2^S of ExTRMs obtained for various values of Outer Square Length during Test Scenario T.S.-80 is shown in Fig. 8a. The MAPE of ExTRMs for various values of Outer Square Length during Test Scenario T.S.-80 is depicted using a comparative bar chart in Fig. 8b. When ExTRMs are trained using first degree PFs, an AR^2^S greater than 0.99 is obtained for all values of Outer Square Length, as shown in Fig. 8a. In addition, the MAPE of the ExTRM is less than 0.47% for all values of Outer Square Length, as shown in Fig. 8b.

Figure 7e–h shows scattergrams of predicted reflectance values vs. simulated reflectance values for Outer Square Length of 24 mm during Test Scenarios T.S.-60, T.S.-70, T.S.-80, and T.S.-90, respectively. Even though only 20% of the simulated data is utilized to forecast the reflectance value for the remaining 80% frequencies, the ExTRM can predict these values with high precision, as shown in Fig. 7g. The same cannot be said for Test Scenario T.S.-90. Supplementary Figs. (S8–S16) shows scattergrams for the remaining Outer Square lengths. As a result, we can conclude that using the ExTRM during antenna design simulation for various values of Outer Square Length can cut simulation requirements by 80%.

The AR^2^S of ExTRMs obtained for various values of Outer Square Length during Test Scenario T.S.-90 is shown in Fig. 8c. The MAPE of ExTRMs for various values of Outer Square Length during Test Scenario T.S.-90 is depicted using a comparative bar chart in Fig. 8d. When ExTRMs are trained using second degree PFs, an AR^2^S of greater than 0.94 can be obtained for all values of Inner Square Length, as shown in Fig. 8c. However, the MAPE of the ExTRM is more than 1.0% for some values of Outer Square Length, as shown in Fig. 8d. As a result, we can conclude that using the ExTRM during antenna design simulation for various values of Outer Square Length cannot cut simulation requirements by 90%.

## Conclusion

To conclude a metamaterial concept based antenna where the patch is truncated and etched with an SRR is simulated and results are verified with the fabricated results. A highly efficient antenna design with high gain, reconfigurable, enhanced reflectance response, and higher bandwidth are proposed. The high gain requirement is met by adding metamaterial superstrates having TWs and SRRs. The reconfigurability is achieved by adding three PIN diode switches. Multiple designs have been observed by adding superstrate layers ranging from one layer to four layers with interchanging TWs and SRRs. The TW metamaterial superstrate design with two layers is giving the best performance of high gain with 15.57 dB, 108 MHz of enhanced bandwidth, − 27.07 dB reflectance response, and six frequency bands. The design is also optimized by changing various physical parameters of the patch design. Machine learning assisted ExTRM is applied to learn the behavior of antenna and predict the reflectance value for a wide range of frequencies. Experimental results prove that the use of the ExTRM based model for simulation of antenna design can cut the simulation time, and resource requirements by 80%. We have developed a smart antenna design using machine learning that can be applied in 5G mobile applications and portable Wi-Fi, Wi-MAX, and WLAN applications.

## Supplementary Information

Supplementary Information.

## Data Availability

The datasets generated and/or analyzed during the current study are available in the github repository, https://github.com/jaymit31/Machine-Learning-assisted-metamaterial-based-reconfigurable-antenna-for-low-cost-portable-electronic].
