# Supplementary material for: Machine learning assisted metamaterial-based reconfigurable antenna for low-cost portable electronic devices
Source: Sci Rep. 2022 Jul 19;12:12354. doi: 10.1038/s41598-022-16678-2 (PMC9296536; doi:10.1038/s41598-022-16678-2)
Supplement: Supplementary file 1 — Supplementary Information. [file 41598_2022_16678_MOESM1_ESM.docx]

**Supplementary Material**

**Machine Learning assisted metamaterial-based reconfigurable antenna for low-cost portable electronic devices**

We have carried out the variation of structural parameters of split ring resonator based patch structure and corresponding results are presented in Fig. S1 and Fig. S2. First, we have varied the outer length of the split ring resonator, X1 in the range of 16 nm to 25 nm with the step of 1 nm, and corresponding reflectance response results are presented for numerous frequency bands for 1 GHz to 7 GHz and for an outer length of 25 nm, we attain excellent results in all the frequency bands of 1.4 GHz to 1.7 GHz, 1.9 GHz to 2.5 GHz, 3.2 GHz to 3.7 GHz, and 4.5 GHz to 5 GHz as reported in Fig. S1(a-d).

Next we have varied the inner length of the split ring resonator, X2 in the range of 10 nm to 15 nm with the step of 1 nm, and corresponding reflectance response results are presented for numerous frequency bands for 1 GHz to 7 GHz and the outer length of 25 nm, we attain excellent results in all the frequency bands of 1.4 GHz to 1.7 GHz, 1.9 GHz to 2.6 GHz, 3.2 GHz to 3.7 GHz, and 4.5 GHz to 5 GHz as reported in Fig. S2(a-d).


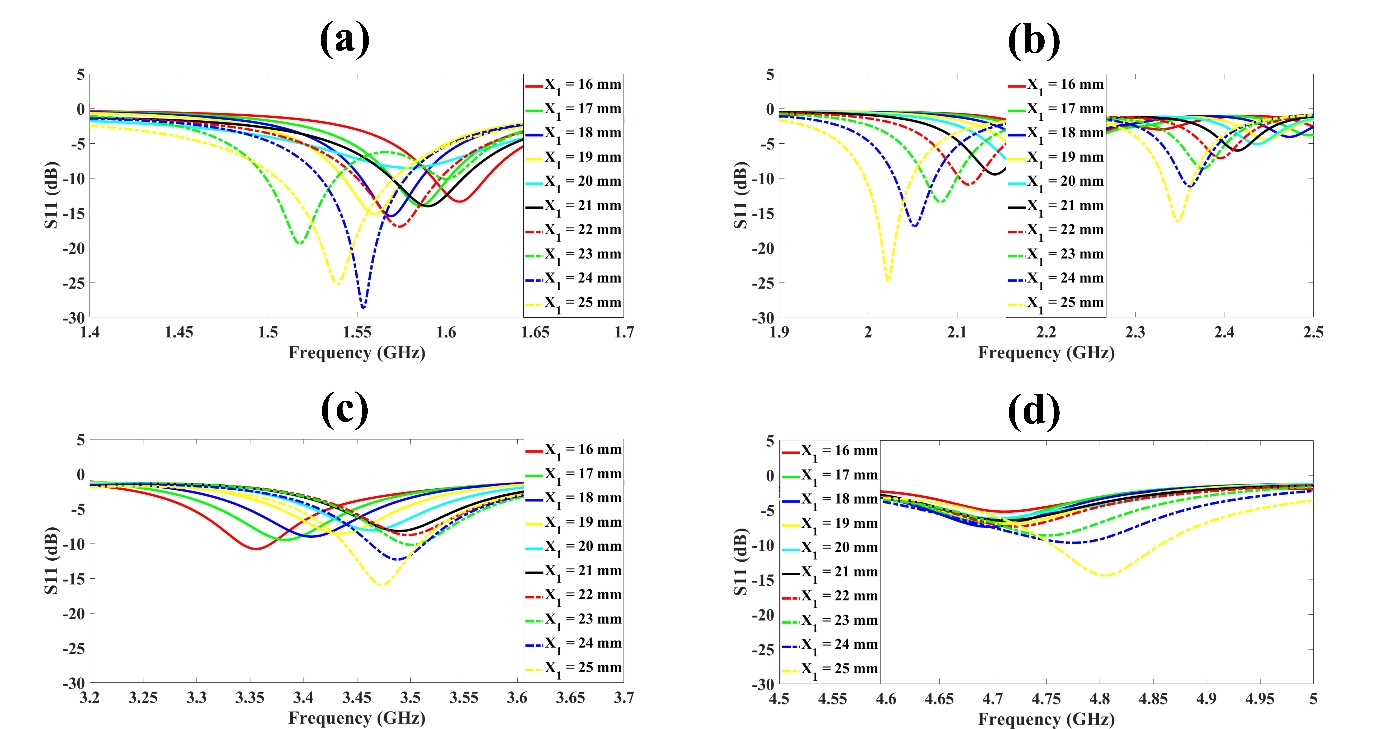


**Fig. S1** Variation of the outer length of split ring patch structure **(a)** for the frequency band of 1.4 GHz to 1.7 GHz, **(b)** for the frequency band of 1.9 GHz to 2.5 GHz, **(c)** for the frequency band of 3.2 GHz to 3.7 GHz, **(d)** for the frequency band of 4.5 GHz to 5 GHz. For X_1_ = 25 mm, we attain a comparatively better reflectance response (>-10 dB) in all the frequency bands. The outer length of the split ring patch structure is varied from 16 mm to 25 mm in the step of 1 mm for the frequency range of 1.4 GHz to 5 GHz.


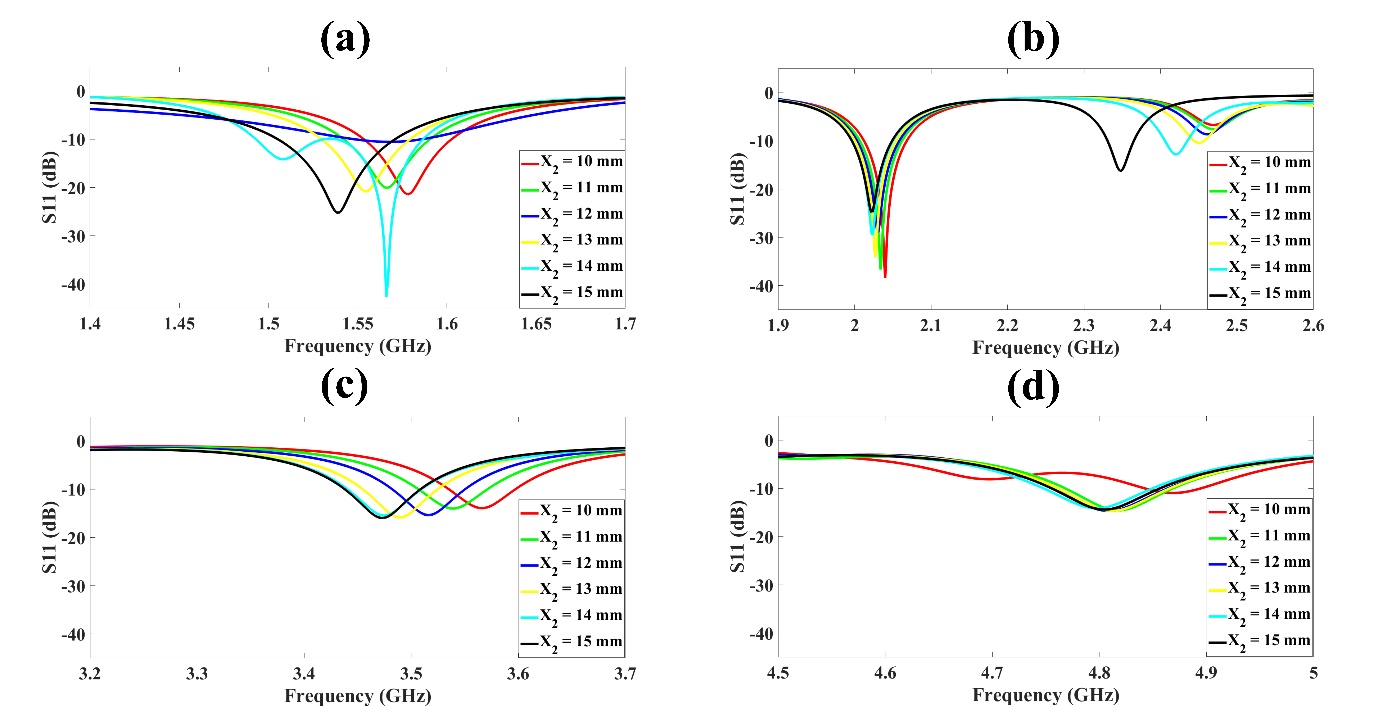


**Fig. S2** Variation of the inner length of split ring patch structure **(a)** for the frequency band of 1.4 GHz to 1.7 GHz, **(b)** for the frequency band of 1.9 GHz to 2.5 GHz, **(c)** for the frequency band of 3.2 GHz to 3.7 GHz, **(d)** for the frequency band of 4.5 GHz to 5 GHz. For X_2_ = 15 mm, we attain a comparatively better reflectance response (>-10 dB) in all the frequency bands. The inner length of the split ring patch structure is varied from 10 mm to 15 mm in the step of 1 mm for the frequency range of 1.4 GHz to 5 GHz.

Supplementary Figure S3 (a-d) shows scattergrams of predicted reflectance values vs. simulated reflectance values for Inner Square Length of 10 mm during Test Scenarios T.S.-60, T.S.-70, T.S.-80, and T.S.-90, respectively. Similarly, supplementary Fig. (S4-S7) shows scattergrams for Inner Square lengths of 11 mm, 12 mm, 13 mm, and 14 mm, respectively.


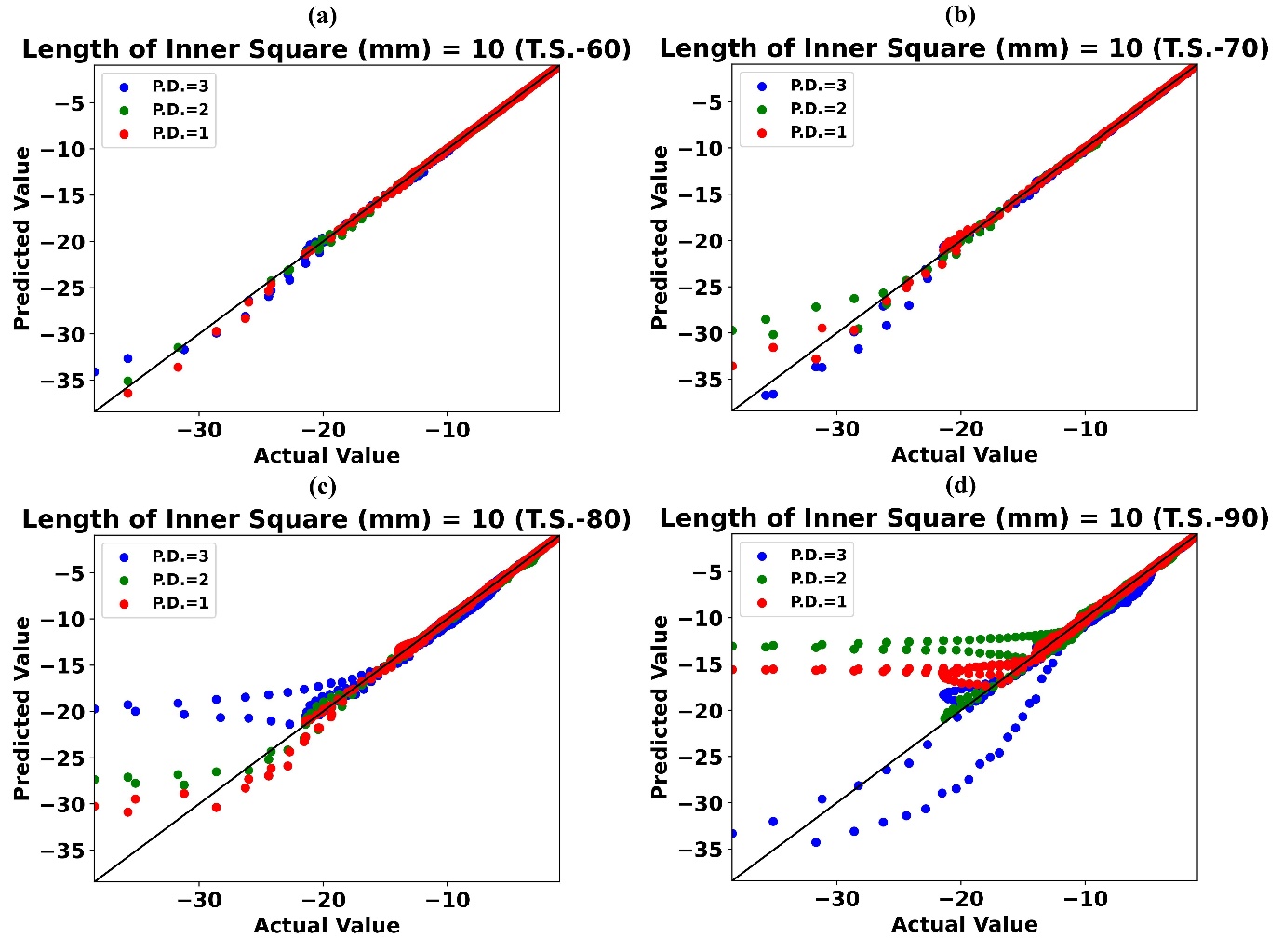


**Fig. S3** Scattergram of Predicted value of reflectance vs Simulated value of reflectance for Length of Inner Square = 10 mm during (a) Test Scenario (T.S.-60) (b) Test Scenario (T.S.-70) (c) Test Scenario (T.S.-80) (d) Test Scenario (T.S.-90)


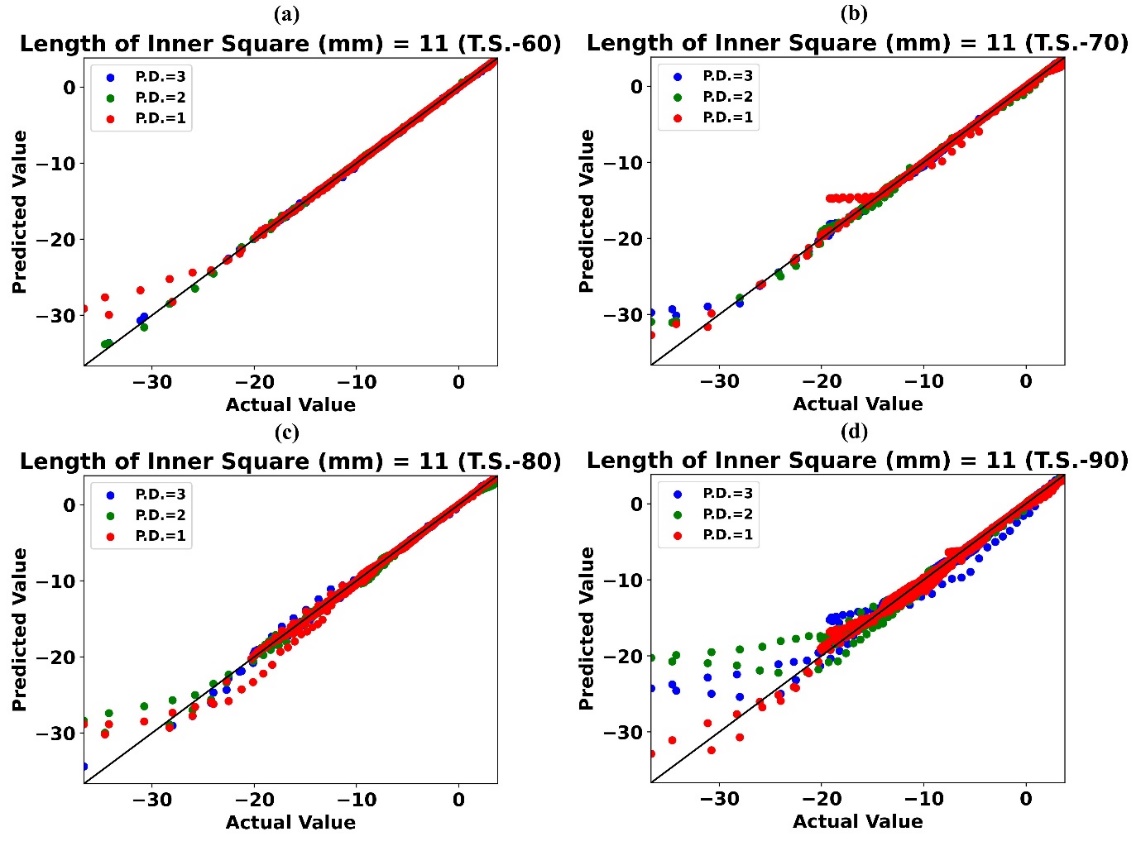


**Fig. S4** Scattergram of Predicted value of reflectance vs Simulated value of reflectance for Length of Inner Square = 11 mm during (a) Test Scenario (T.S.-60) (b) Test Scenario (T.S.-70) (c) Test Scenario (T.S.-80) (d) Test Scenario (T.S.-90)


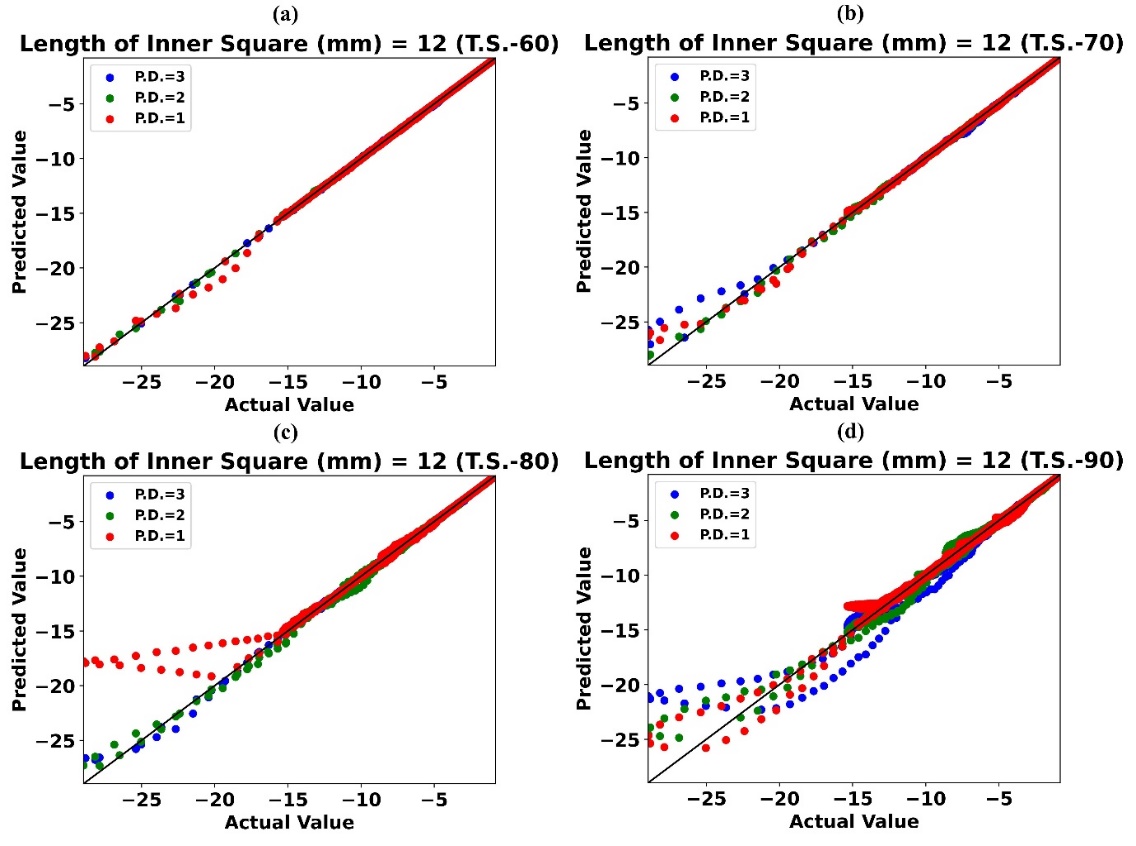


**Fig. S5** Scattergram of Predicted value of reflectance vs Simulated value of reflectance for Length of Inner Square = 12 mm during (a) Test Scenario (T.S.-60) (b) Test Scenario (T.S.-70) (c) Test Scenario (T.S.-80) (d) Test Scenario (T.S.-90)


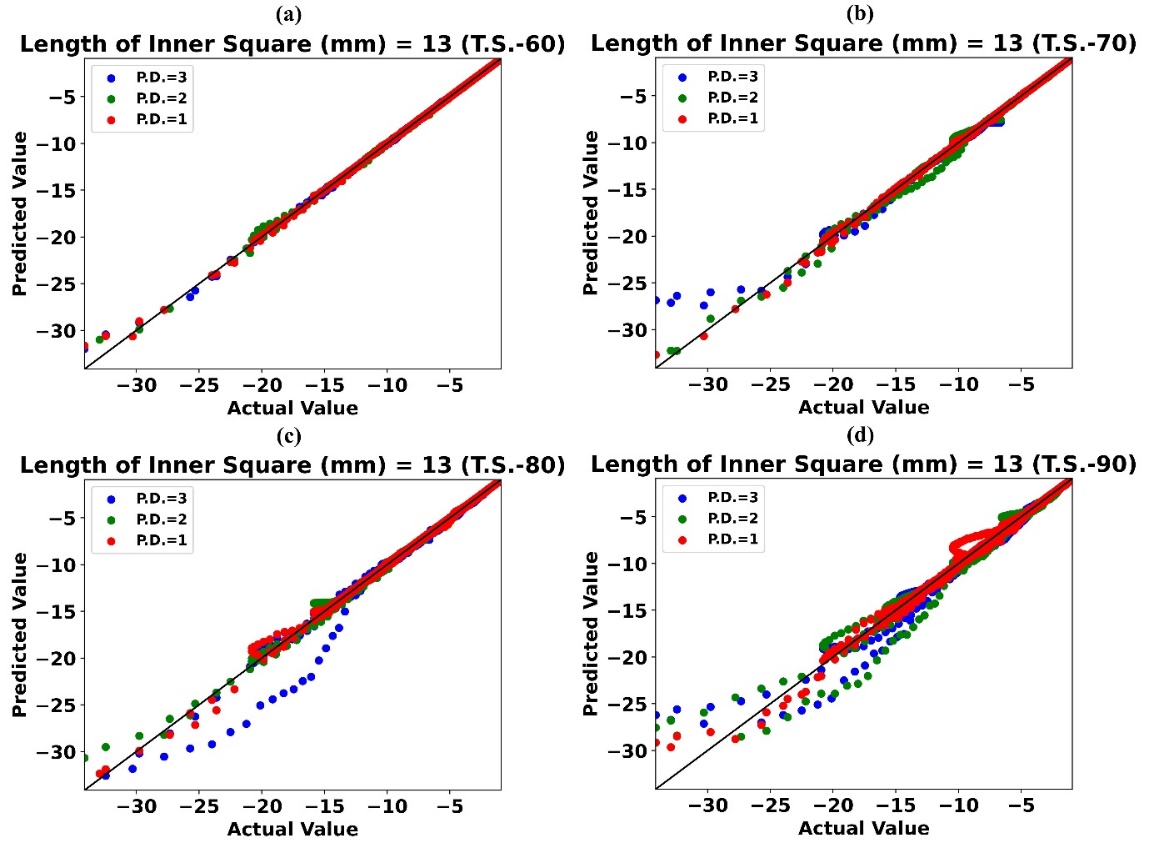


**Fig. S6** Scattergram of Predicted value of reflectance vs Simulated value of reflectance for Length of Inner Square = 13 mm during (a) Test Scenario (T.S.-60) (b) Test Scenario (T.S.-70) (c) Test Scenario (T.S.-80) (d) Test Scenario (T.S.-90)


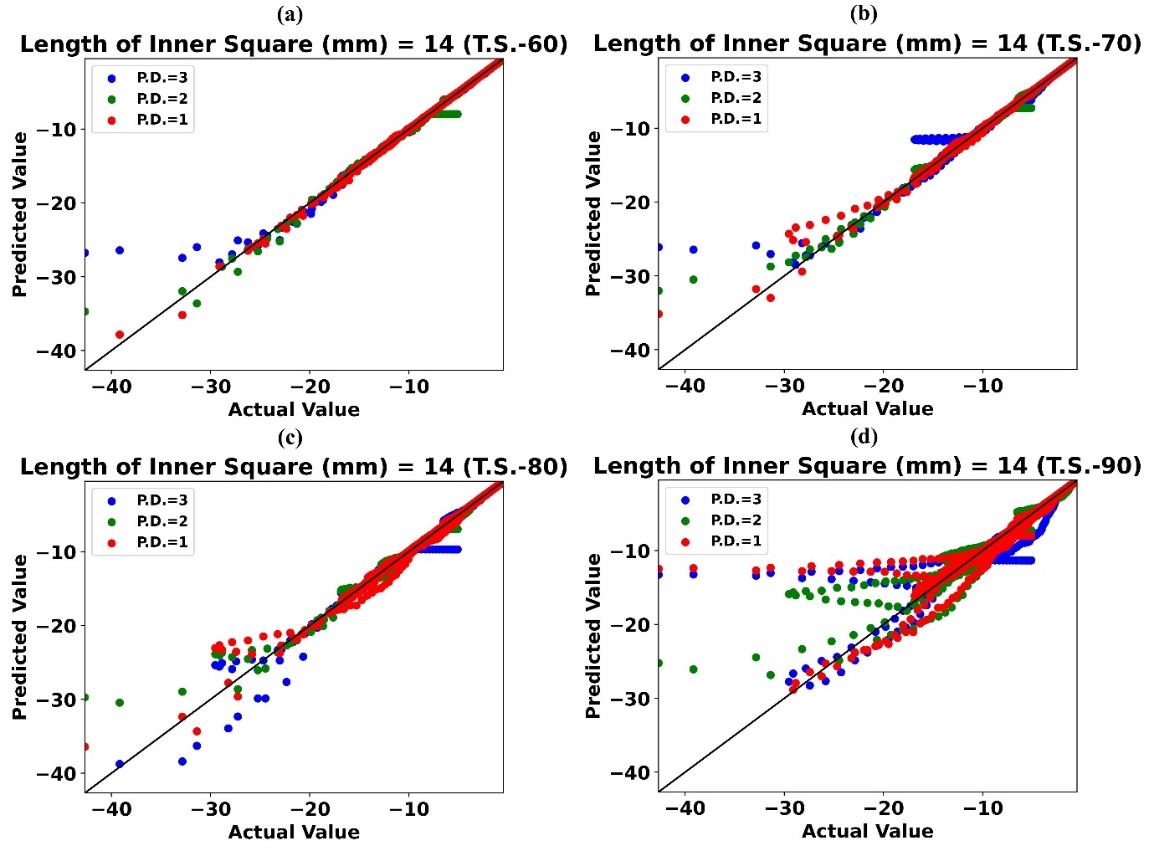


**Fig. S7** Scattergram of Predicted value of reflectance vs Simulated value of reflectance for Length of Inner Square = 14 mm during (a) Test Scenario (T.S.-60) (b) Test Scenario (T.S.-70) (c) Test Scenario (T.S.-80) (d) Test Scenario (T.S.-90)

Supplementary Figure S8 (a-d) shows scattergrams of predicted reflectance values vs. simulated reflectance values for Outer Square Length of 15 mm during Test Scenarios T.S.-60, T.S.-70, T.S.-80, and T.S.-90, respectively. Similarly, supplementary Fig. (S9-S16) shows scattergrams for Outer Square lengths of 16 mm, 17 mm, 18 mm, 19 mm, 20 mm, 21 mm, 22 mm, 23 mm, 24 mm respectively.


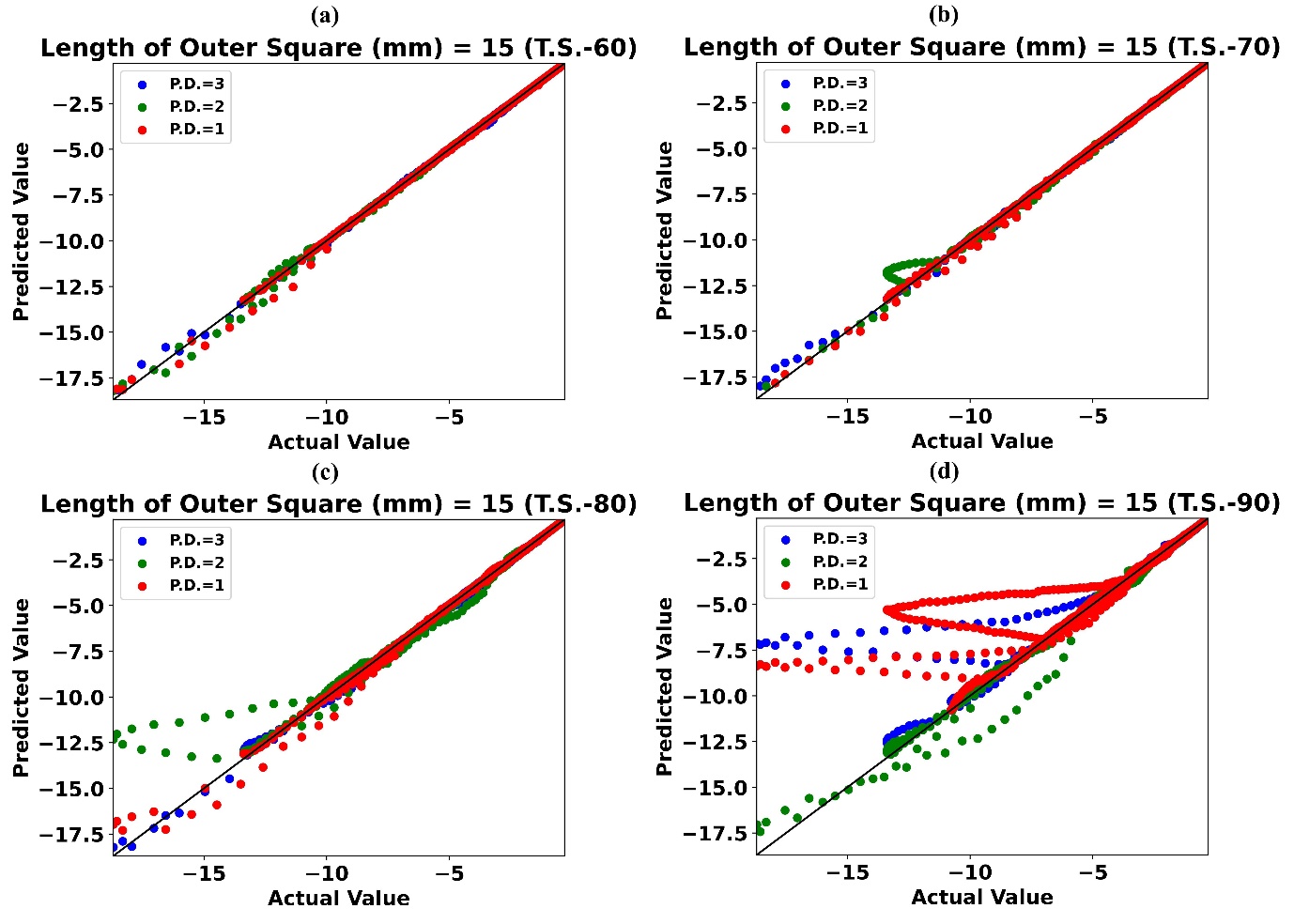


**Fig. S8** Scattergram of Predicted value of reflectance vs Simulated value of reflectance for Length of Outer Square = 15 mm during (a) Test Scenario (T.S.-60) (b) Test Scenario (T.S.-70) (c) Test Scenario (T.S.-80) (d) Test Scenario (T.S.-90)


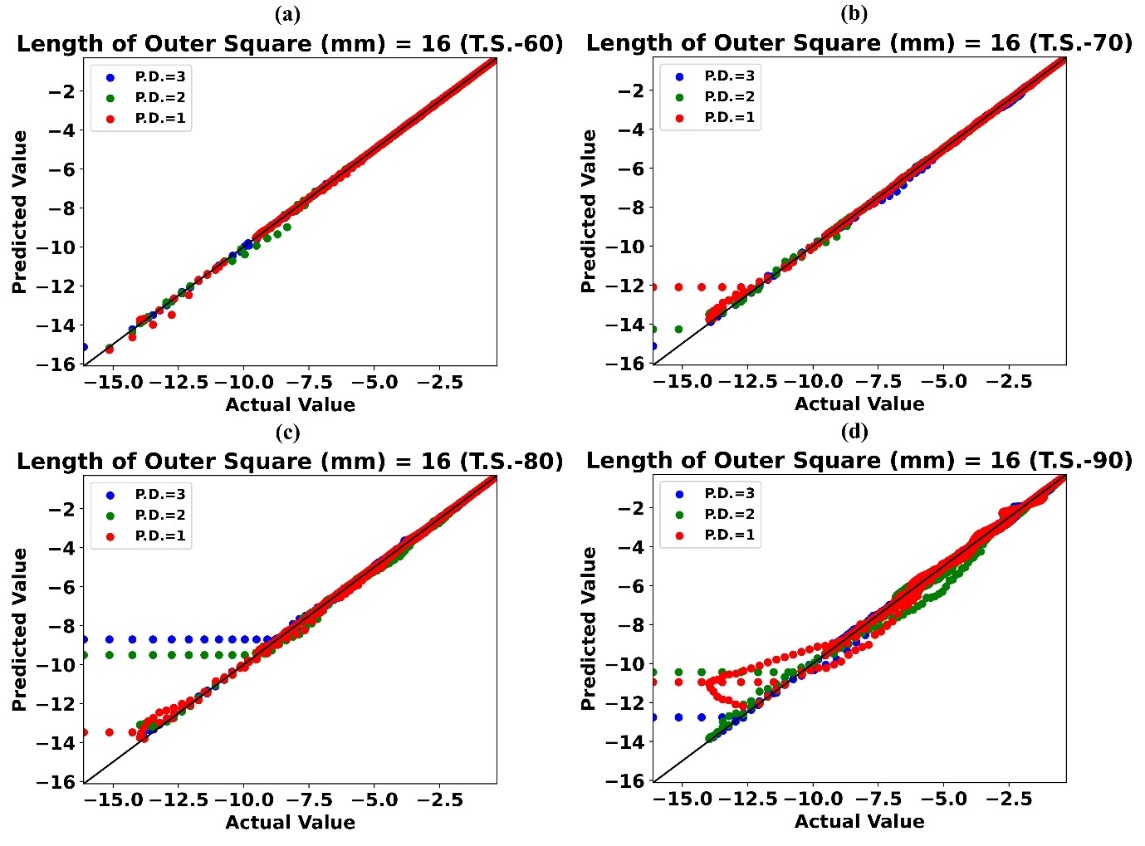


**Fig. S9** Scattergram of Predicted value of reflectance vs Simulated value of reflectance for Length of Outer Square = 16 mm during (a) Test Scenario (T.S.-60) (b) Test Scenario (T.S.-70) (c) Test Scenario (T.S.-80) (d) Test Scenario (T.S.-90)


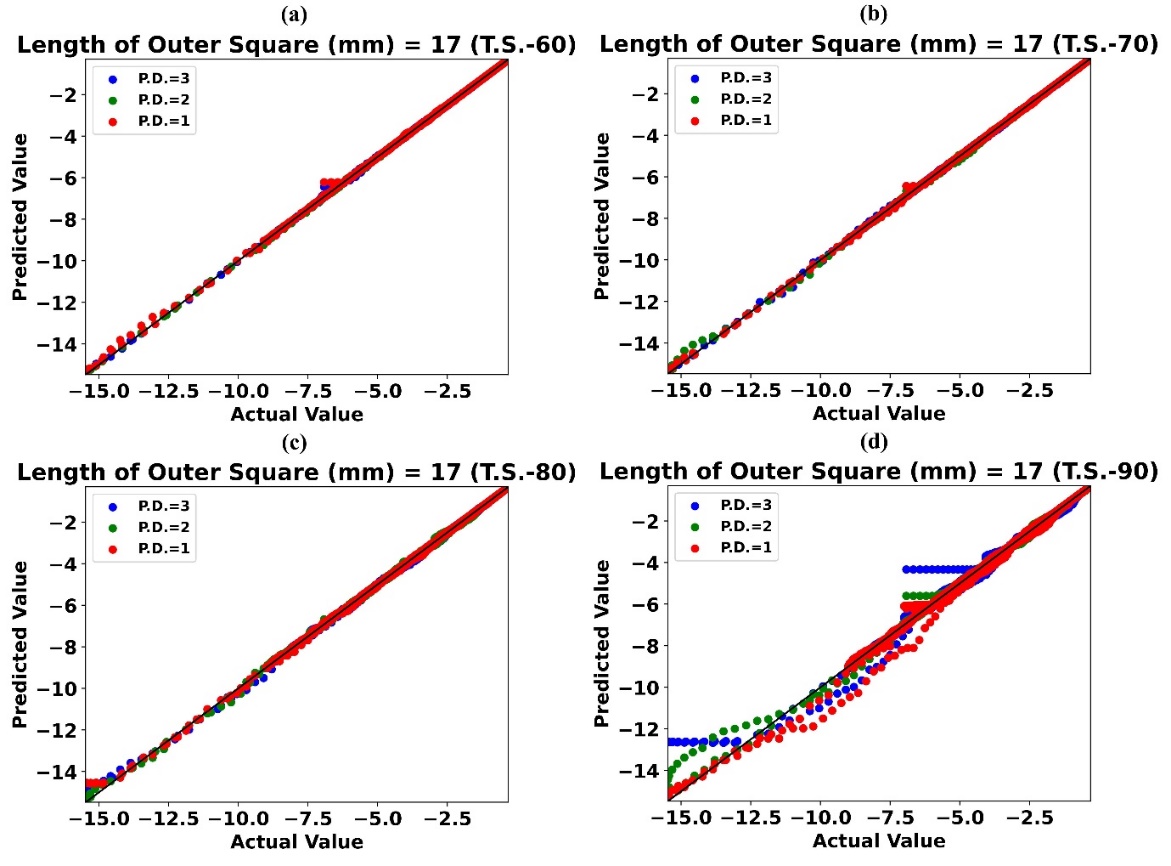


**Fig. S10** Scattergram of Predicted value of reflectance vs Simulated value of reflectance for Length of Outer Square = 17 mm during (a) Test Scenario (T.S.-60) (b) Test Scenario (T.S.-70) (c) Test Scenario (T.S.-80) (d) Test Scenario (T.S.-90)


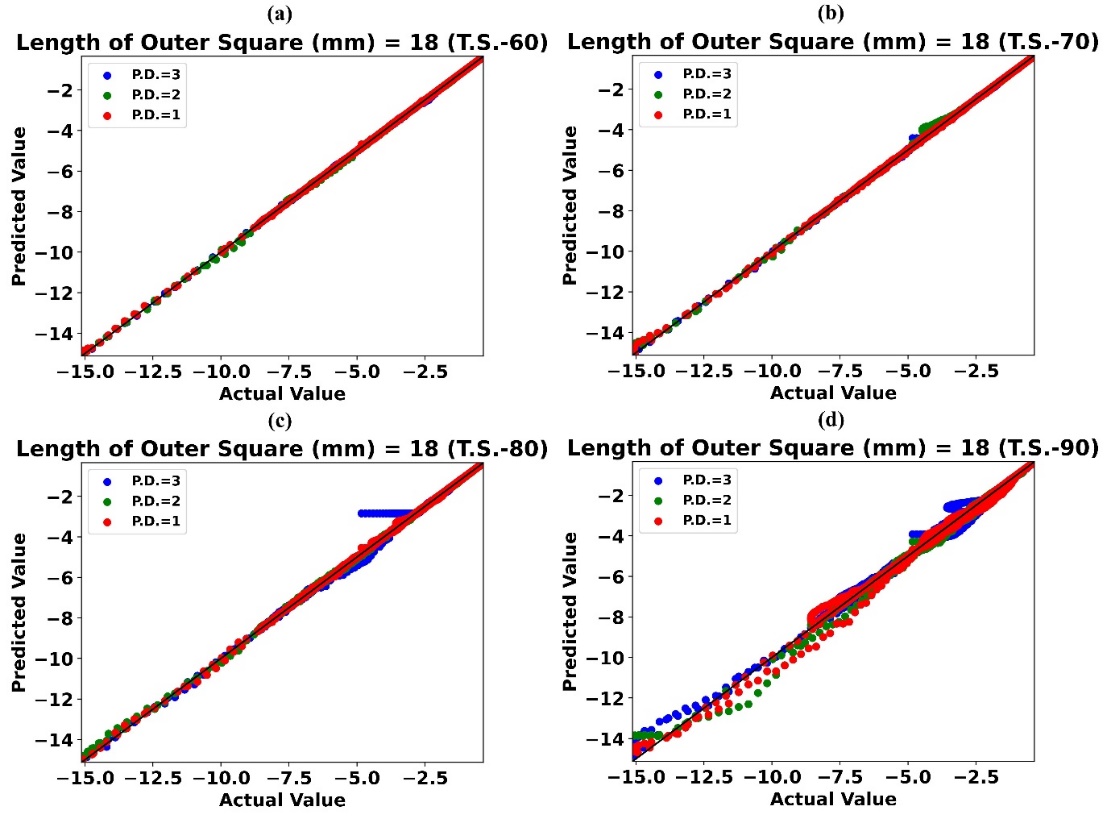


**Fig. S11** Scattergram of Predicted value of reflectance vs Simulated value of reflectance for Length of Outer Square = 18 mm during (a) Test Scenario (T.S.-60) (b) Test Scenario (T.S.-70) (c) Test Scenario (T.S.-80) (d) Test Scenario (T.S.-90)


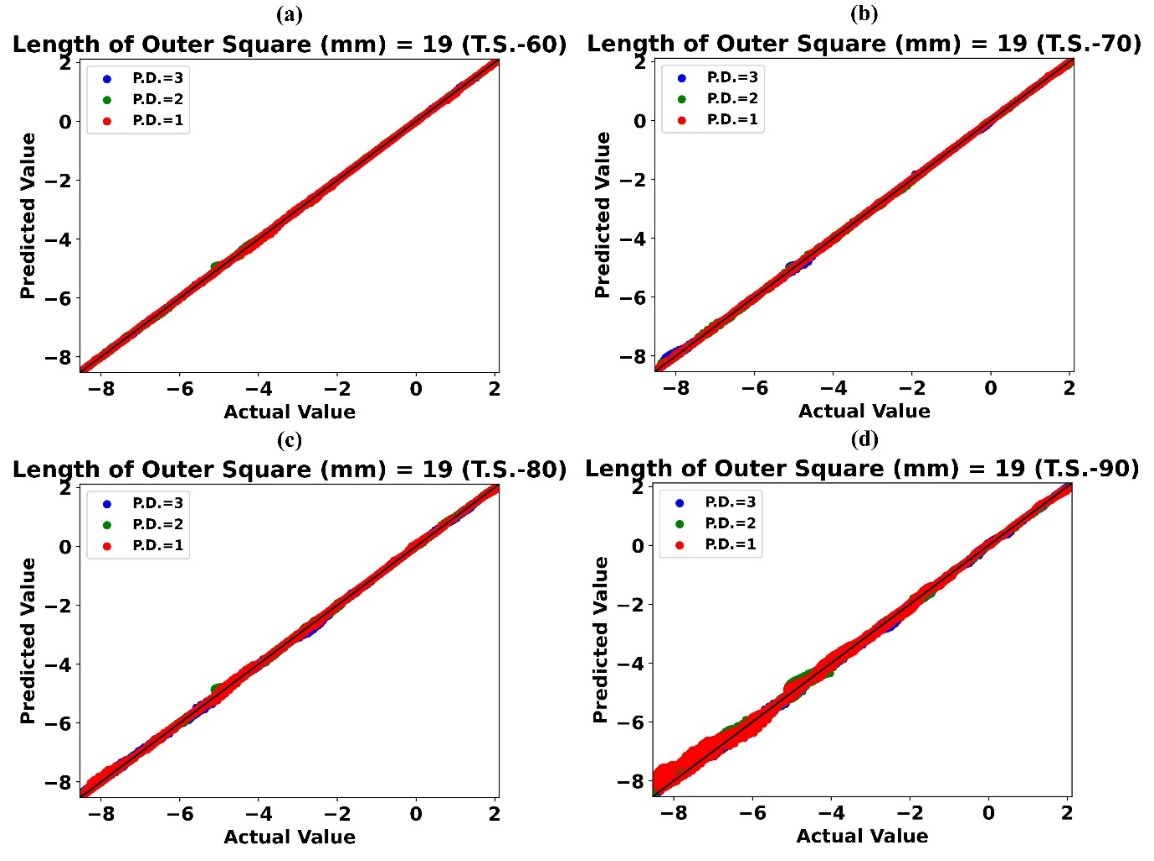


**Fig. S12** Scattergram of Predicted value of reflectance vs Simulated value of reflectance for Length of Outer Square = 19 mm during (a) Test Scenario (T.S.-60) (b) Test Scenario (T.S.-70) (c) Test Scenario (T.S.-80) (d) Test Scenario (T.S.-90)


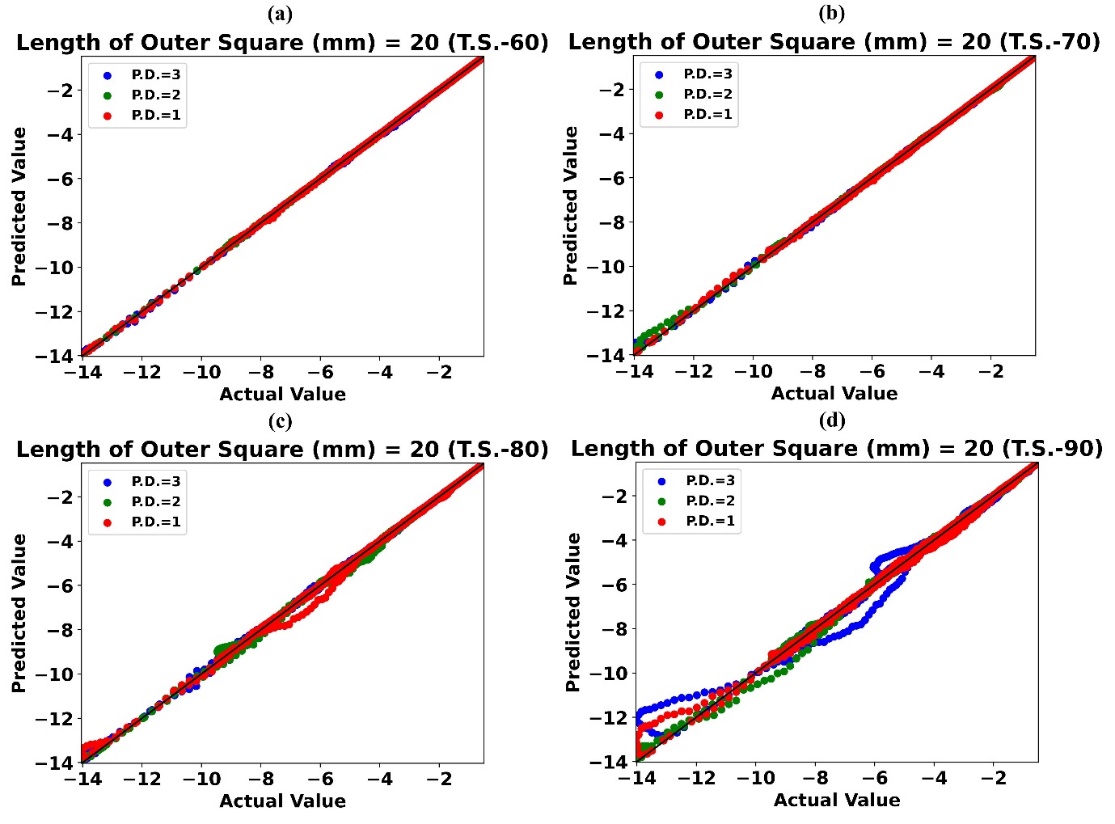


**Fig. S13** Scattergram of Predicted value of reflectance vs Simulated value of reflectance for Length of Outer Square = 20 mm during (a) Test Scenario (T.S.-60) (b) Test Scenario (T.S.-70) (c) Test Scenario (T.S.-80) (d) Test Scenario (T.S.-90)


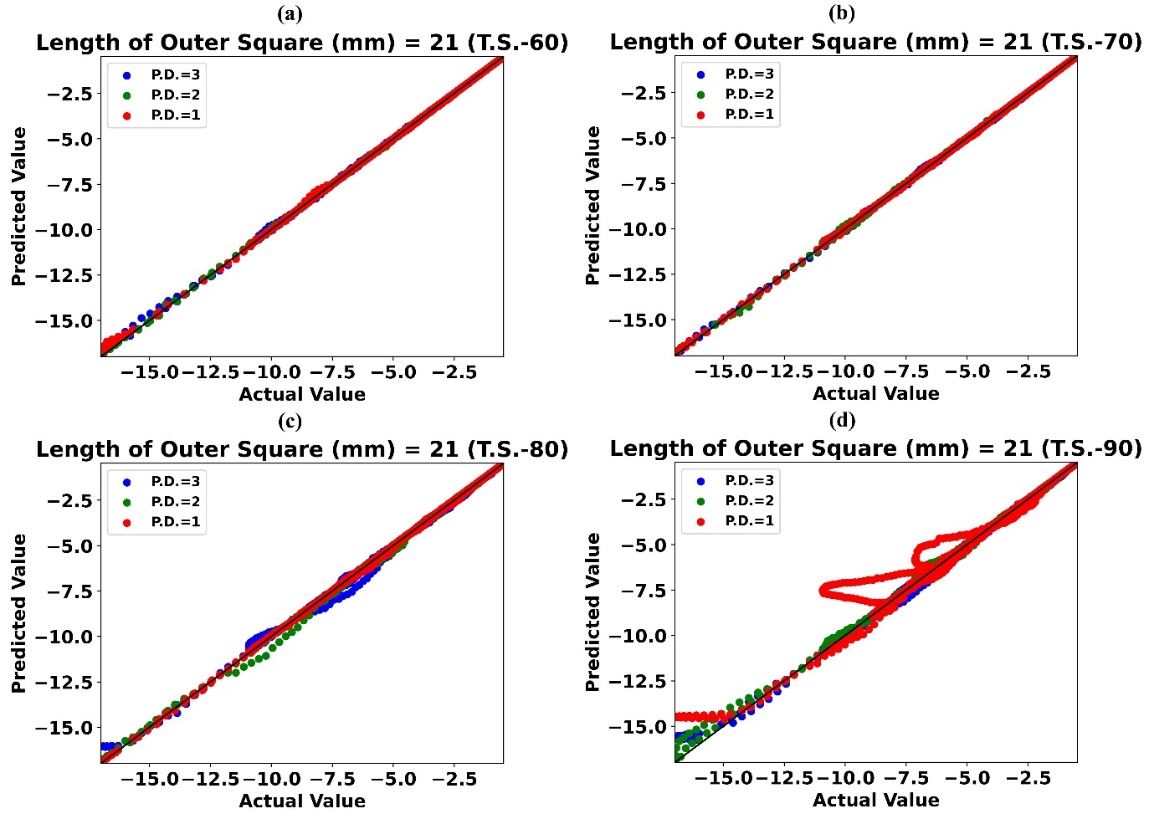


**Fig. S14** Scattergram of Predicted value of reflectance vs Simulated value of reflectance for Length of Outer Square = 21 mm during (a) Test Scenario (T.S.-60) (b) Test Scenario (T.S.-70) (c) Test Scenario (T.S.-80) (d) Test Scenario (T.S.-90)


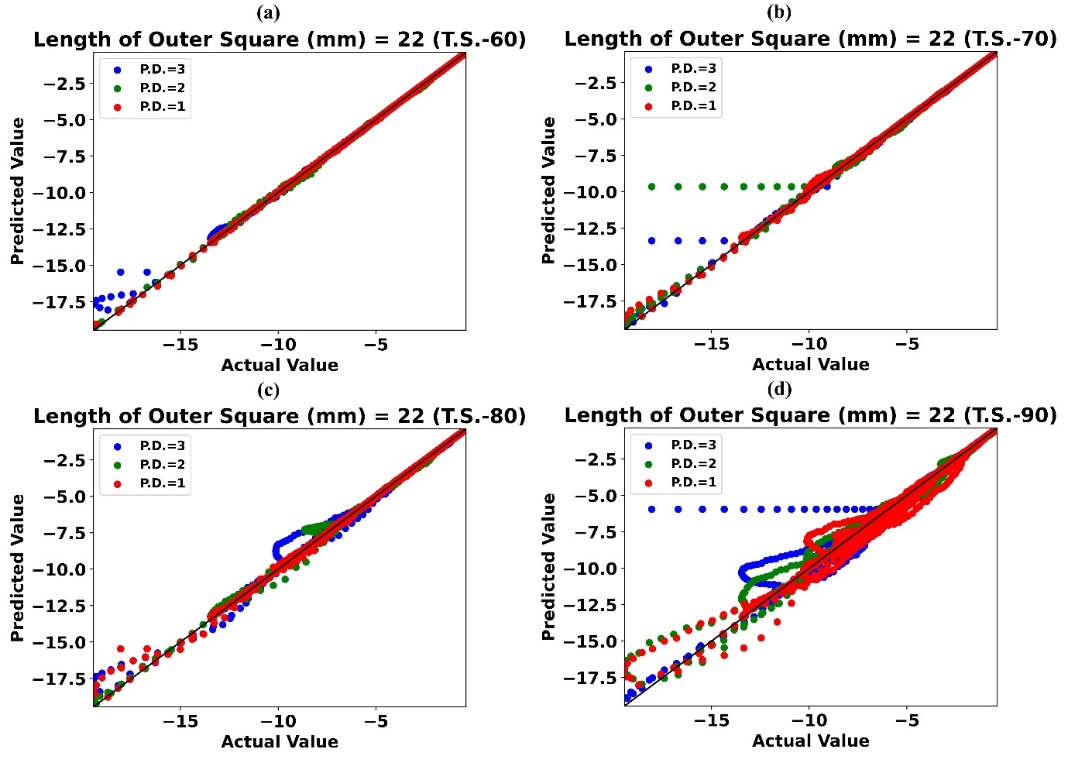


**Fig. S15** Scattergram of Predicted value of reflectance vs Simulated value of reflectance for Length of Outer Square = 22 mm during (a) Test Scenario (T.S.-60) (b) Test Scenario (T.S.-70) (c) Test Scenario (T.S.-80) (d) Test Scenario (T.S.-90)


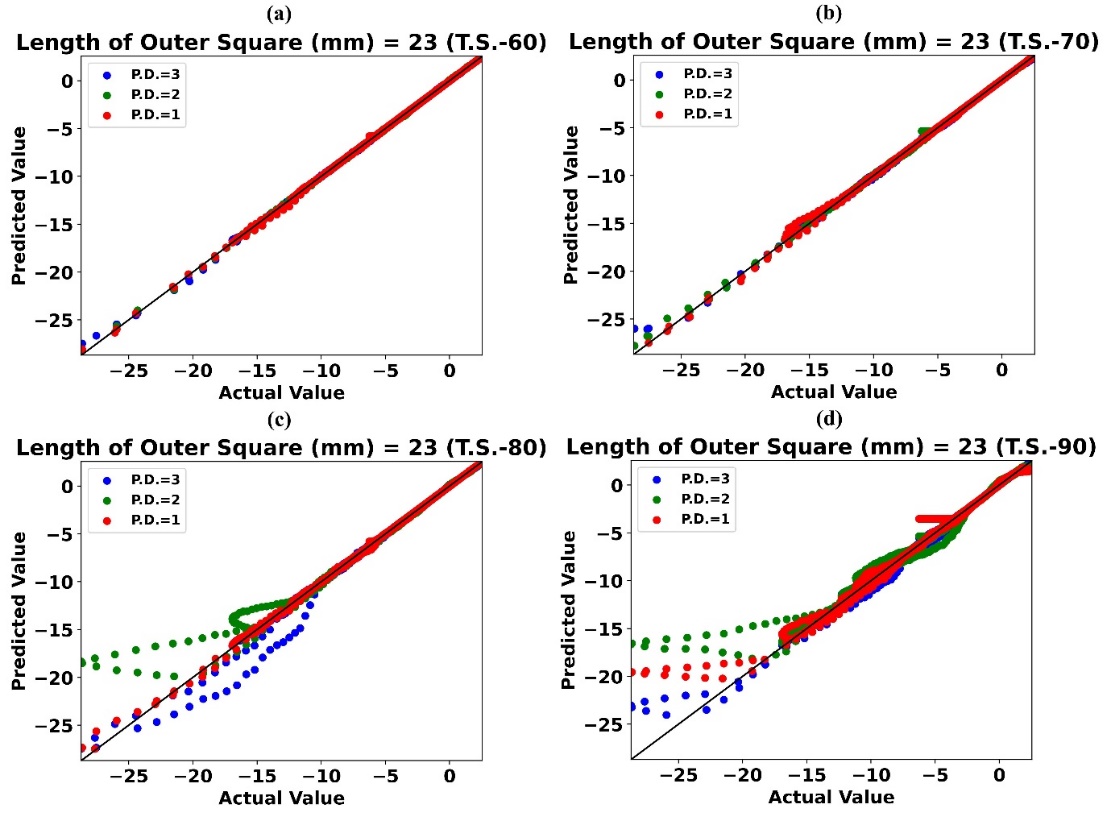


**Fig. S16** Scattergram of Predicted value of reflectance vs Simulated value of reflectance for Length of Outer Square = 23 mm during (a) Test Scenario (T.S.-60) (b) Test Scenario (T.S.-70) (c) Test Scenario (T.S.-80) (d) Test Scenario (T.S.-90)

**Fig. S17** (a) The Conformation of RF PIN diode model for HFSS tool (b) ON state of the switch. (c) OFF state of the switch [1]

RF switches can be made most easily with the PIN diode [1]. Figure S17(a) shows the HFSS equivalent model of a PIN diode. A PIN diode's ON and OFF states are shown in Figs. S17(b) and S17(c), respectively, to show the corresponding lumped components resistors (R_s_) and capacitors (C_p_) in series and in parallel, respectively, with an inductor (L_s_) (Fig. S17(a)).


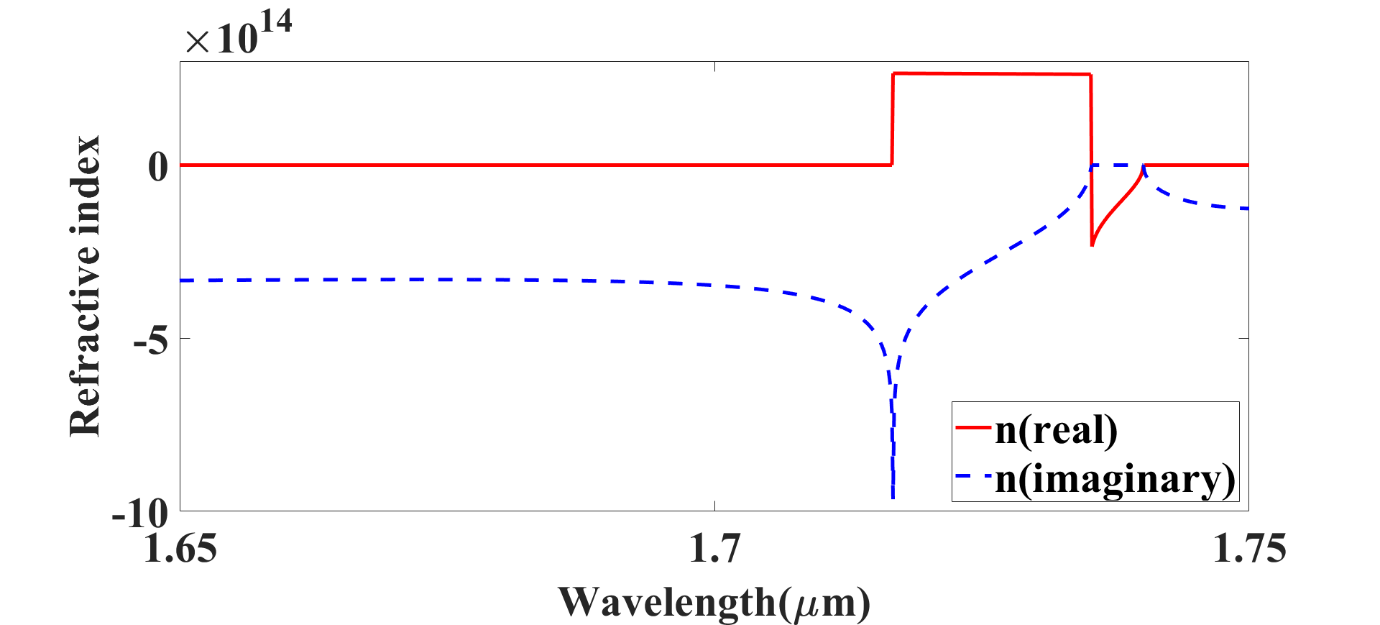


**Fig. S18** Refractive index of single layer 6 × 5 thin wire structure

The refractive index plot for single layer 6 × 5 thin wire structure is presented in Fig. S18 and we achieved a negative refractive index at around 1.74 GHz. We have only provided the plot fore this one structure from eq. (1-4) and in the same manner we can calculate the rest of quantities for other designs. The negative refractive index is one of the major property of metamaterial and achieving it validates the metamaterial behaviour of the proposed structure.

A comparison study to compare the proposed antenna structures with previously published work is also carried out and presented in supplementary Table ST1.

**Table ST1.** Comparison table of proposed antenna structures with previously published work

| Antenna Structure | Reflectance Response, S_11_ (dB) | No. of Bands | Bandwidth  (MHz) | Gain (dB) |
| --- | --- | --- | --- | --- |
| 6 × 5 thin wire single layer structure | -24.05 | 5 | 100 | 3.58 |
| **6 × 5 thin wire double layer structure** | **-27.07** | **6** | **108** | **15.57** |
| 6 × 5 thin wire triple layer structure | -27.18 | 5 | 105 | 12.37 |
| 6 × 5 thin wire four layer structure | -23.74 | 5 | 105 | 7.71 |
| Split ring resonator (SRR) four layer structure | -27.40 | 5 | 105 | 8.49 |
| Split ring resonator three layer combined with 6 × 5 thin wire single layer as a top layer | -41.97 | 5 | 110 | 1.49 |
| Ref. [2] | - | - | 290 | 2.6 |
| Ref. [3] | - | - | 180 | 3 |
| Ref. [4] | - | - | - | 5.08 |
| Ref. [5] | - | - | 400 | - |
| Ref. [6] | - | - | 8800 | 4.1 |
| Ref. [7] | - | - | 4300 | 6.8 |

**Table ST2: Details of Test Scenarios used to quantity the prediction correctness of trained regression model**

| Test Scenario | No. of Training Samples | No of Testing Samples |
| --- | --- | --- |
| T.S.-60 | 24,000 | 36,000 |
| T.S.-70 | 18,000 | 42,000 |
| T.S.-80 | 12,000 | 48,000 |
| T.S.-90 | 6000 | 54,000 |

**References**

[1] K.Sumathi, S. Lavadiya, P. Z. Yin, J. Parmar, and S. K. Patel, “High gain multiband and frequency reconfigurable metamaterial superstrate microstrip patch antenna for C/X/Ku-band wireless network applications,” *Wirel. Networks*, 2021, doi: 10.1007/s11276-021-02567-5.

[2] P. K. Li, Z. H. Shao, Q. Wang, and Y. J. Cheng, “Frequency- and pattern-reconfigurable antenna for multistandard wireless applications,” *IEEE Antennas Wirel. Propag. Lett.*, vol. 14, pp. 333–336, 2015, doi: 10.1109/LAWP.2014.2359196.

[3] Y. P. Selvam, L. Elumalai, M. G. N. Alsath, M. Kanagasabai, S. Subbaraj, and S. Kingsly, “Novel Frequency- A nd Pattern-Reconfigurable Rhombic Patch Antenna with Switchable Polarization,” *IEEE Antennas Wirel. Propag. Lett.*, vol. 16, pp. 1639–1642, 2017, doi: 10.1109/LAWP.2017.2660069.

[4] R. Dewan, M. K. A. Rahim, M. R. Hamid, M. Himdi, H. A. Majid, and N. A. Samsuri, “HIS-EBG unit cells for pattern and frequency reconfigurable dual band array antenna,” *Prog. Electromagn. Res. M*, vol. 76, pp. 123–132, 2018, doi: 10.2528/PIERM18090202.

[5] L. Han, C. Wang, W. Zhang, R. Ma, and Q. Zeng, “Design of frequency- and pattern-reconfigurable wideband slot antenna,” *Int. J. Antennas Propag.*, vol. 2018, 2018, doi: 10.1155/2018/3678018.

[6] G. Jin, M. Li, D. Liu, and G. Zeng, “A Simple Planar Pattern-Reconfigurable Antenna Based on Arc Dipoles,” *IEEE Antennas Wirel. Propag. Lett.*, vol. 17, no. 9, pp. 1664–1668, 2018, doi: 10.1109/LAWP.2018.2862624.

[7] B. Ashvanth, B. Partibane, M. G. Nabi Alsath, and R. Kalidoss, “Tunable dual band antenna with multipattern reconfiguration for vehicular applications,” *Int. J. RF Microw. Comput. Eng.*, vol. 29, no. 12, 2019, doi: 10.1002/mmce.21973.
